# Supplementary material for: Slc11 Synapomorphy: A Conserved 3D Framework Articulating Carrier Conformation Switch
Source: Int J Mol Sci. 2023 Oct 11;24(20):15076. doi: 10.3390/ijms242015076 (PMC10606218; doi:10.3390/ijms242015076)
Supplement: Supplementary file 1 [file ijms-24-15076-s001.zip › ijms-2587053-supplementary figures.pdf]

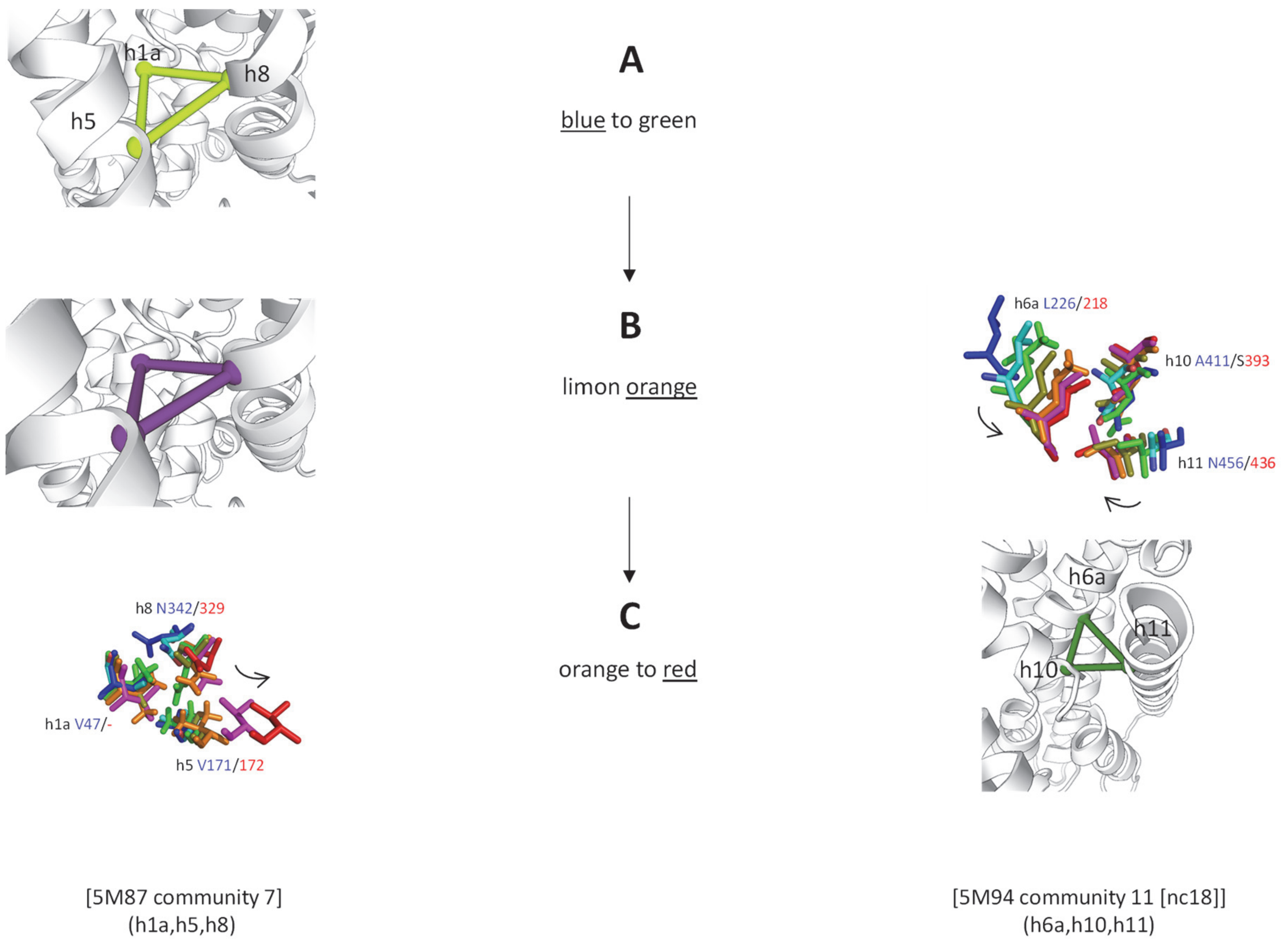

Figure S1. Alternate communities of networked residues distinguish MCb OO state from IO state. *Left panel*. h8 N342/329 (5M87/5M94) is part of a small network connecting to h1a and h5. This network holds during initial progression through OO state (blue to green, cf Figure 2, **A**); it evolves during intermediate steps of conformational exchange (switch) between OO and IO states (limon and orange, **B**) before disruption, by subsequent displacement of h5 while advancing through IO state (magenta and red, **C**). *Right panel*. Another small network on the opposite side of the molecule connects only in later steps of OO to IO transition, i.e., terminating the conformation switch (post-limon intermediate, **C**).

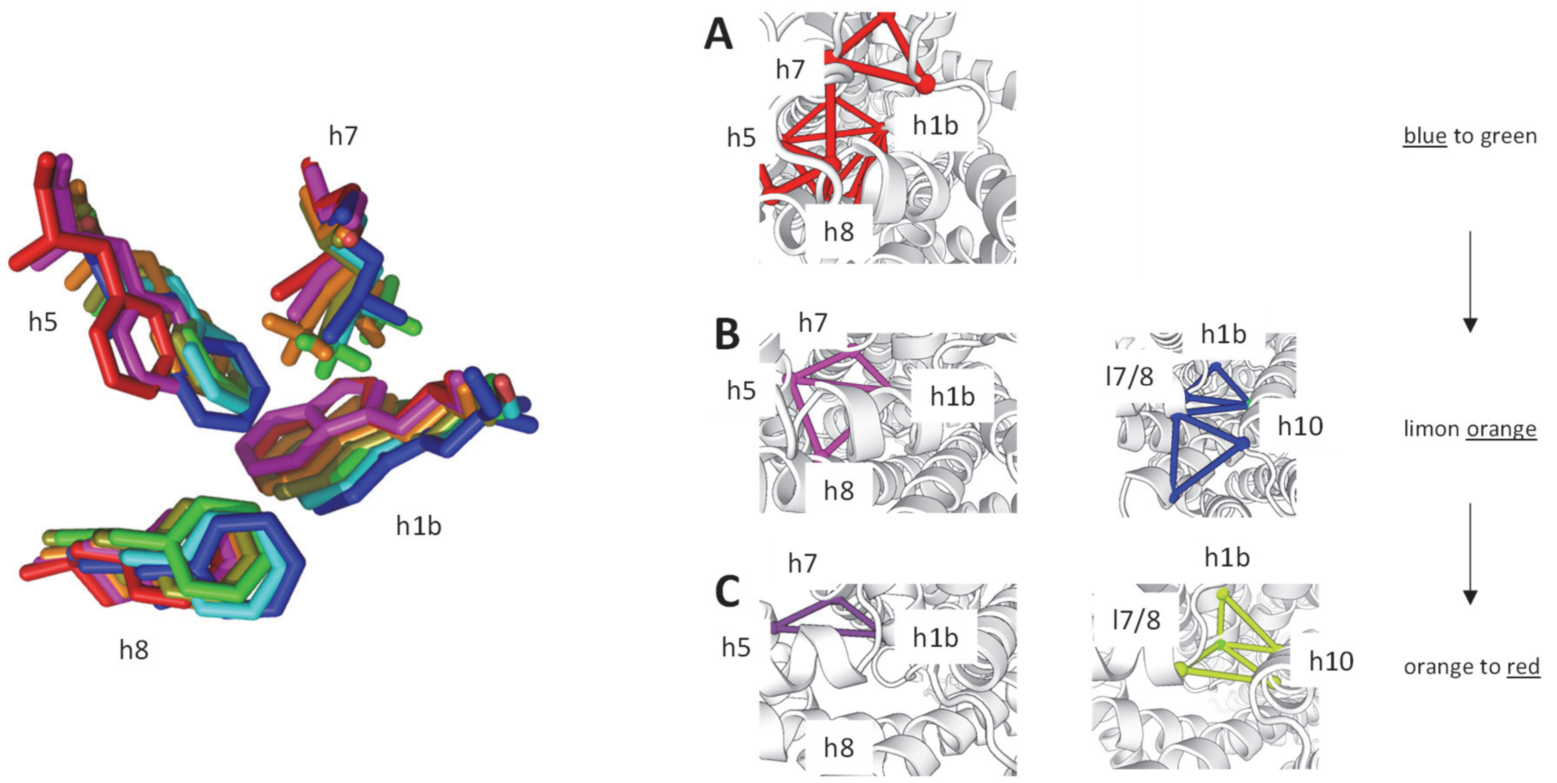

Figure S2. Community of hydrophobic residues evolving during MCb transition from OO state to IO state. *Left panel.* Relative side chain orientation and position of the four-residue community linking h1b, h5, h7 and h8. *Right panel.* **A**. This community is part of a larger network through the ensemble of blue to green OO models. **B**. The four-residue community is maintained in the pair of limon-orange models together with neo connections linking h1b to I7/8 and h10. **C**. Only three residues remain connected in the ensemble of orange to red IO models (minus h8 F) also with additional connections between h1b, l7/8 and h10.

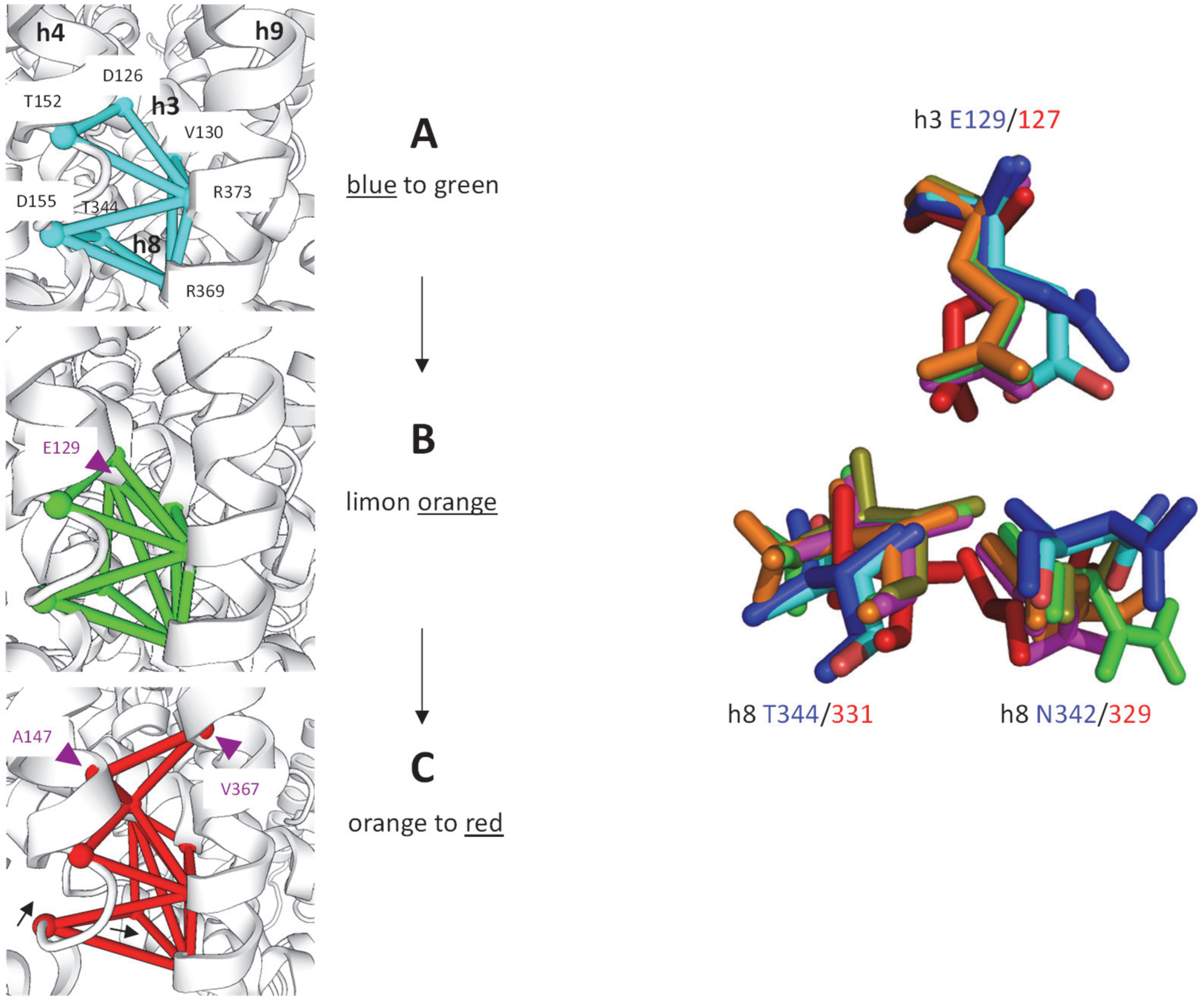

Figure S3. MCb H$^+$-network evolves during MCb transition from OO state to IO state. *Left panel*. h3 E129/127 (5M87/5M94) is connected to the H$^+$ network (including h8 T344/331) in the limon intermediate model (**A**, **B**). Topological reorganization of this network between h4 and h9 later coincides with inner gate opening (ensemble of orange to red models, black arrows, **C**). *Right panel*. h8 N342/329 seems continuously moving throughout carrier OO to IO transition (cf per residue RMSD plots, Figure 3) and remains connected to h1a and h5 until late OO steps (cf Figure S1, left panel).

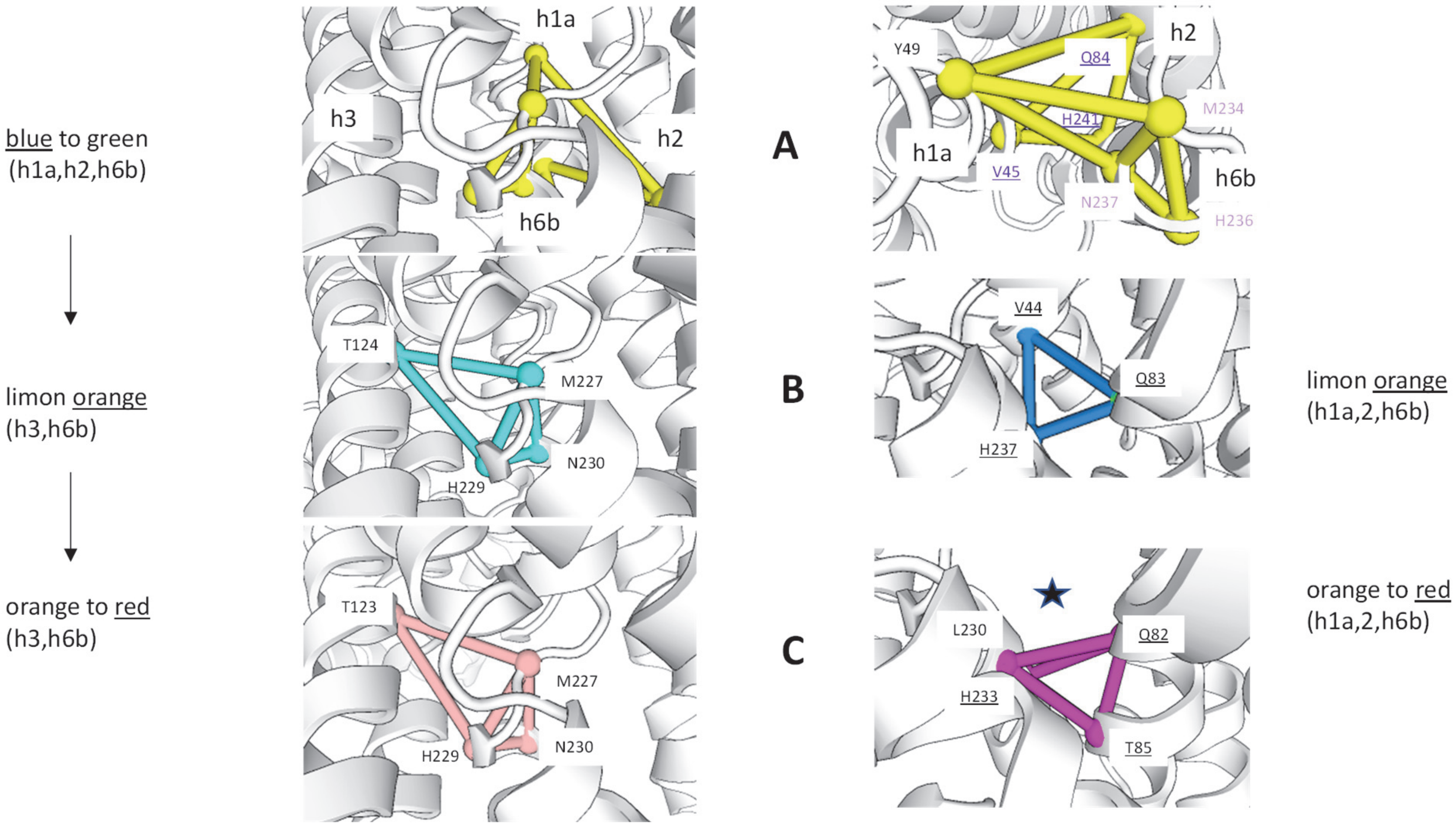

Figure S4. Alternate h6b connections during MCb forward conformation switch (OO to IO). **A**. In OO state (blue to green model ensemble) h6b and h1a are connected both at their top and bottom (cf right panel). **B**, **C**. This community of networked residues splits in two during OO to IO conformation switch (pair of limon orange models). A star marks the absence of h1a from 5M94 structure (**C**).

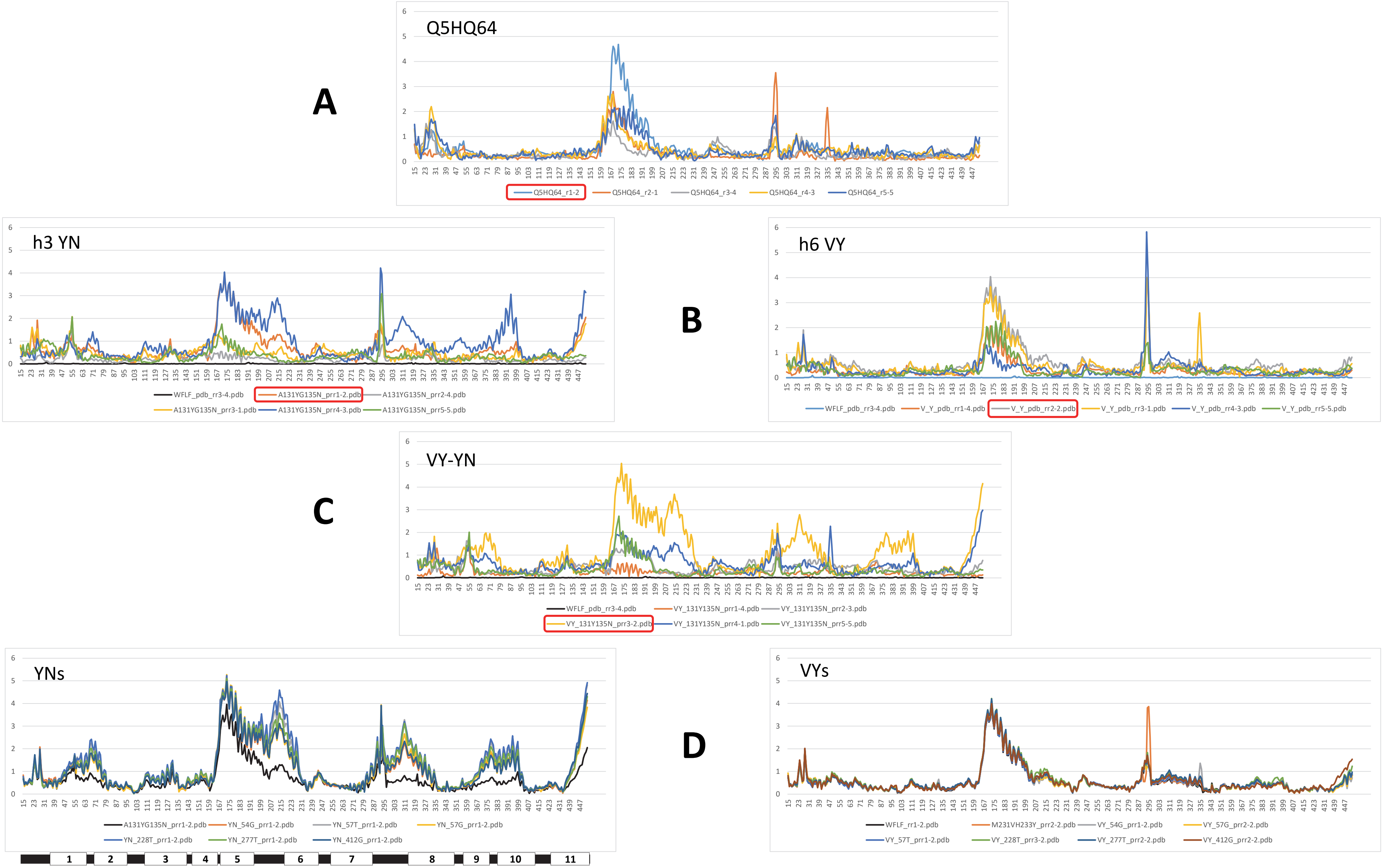

Figure S5. Choice of AF2 trained model2 output (of CF modeling with pdb template) for MCb Q5HQ64 mutagenesis study. **A**. Per residue RMSD of 5 Q5HQ64 CF pdb models each structurally aligned with AF2 Q5HQ64 reference model. Model2, highlighted in red, ranks 1 and appears prone to locally rearrange wt Q5HQ64, with limited inner gate (h4/h5) opening compared to AF2 IO model; Model4 was chosen as baseline reference for subsequent pairwise alignment of mutant Q5HQ64 models. **B-D**. Per residue RMSD of CF pdb models for various Q5HQ64 mutants structurally aligned onto native Model4. 5 CF pdb models per mutant: h3 YN (A131Y, G135N) or h6 VY (M231V, H233Y) (**B**), h3 YN h6 VY (**C**), with model2 highlighted in red. CF pdb model2 for compound mutants combining h3 YN or h6 VY with point mutations in helices h1 (D54G, N57T, N57G), h6 (A228T), h7 (N277T) and h11 (N442G) (**D**). None of the latter mutations in h1, h6, h7 or h11 was sufficient alone to induce broad scale change in RMSD (data not shown).

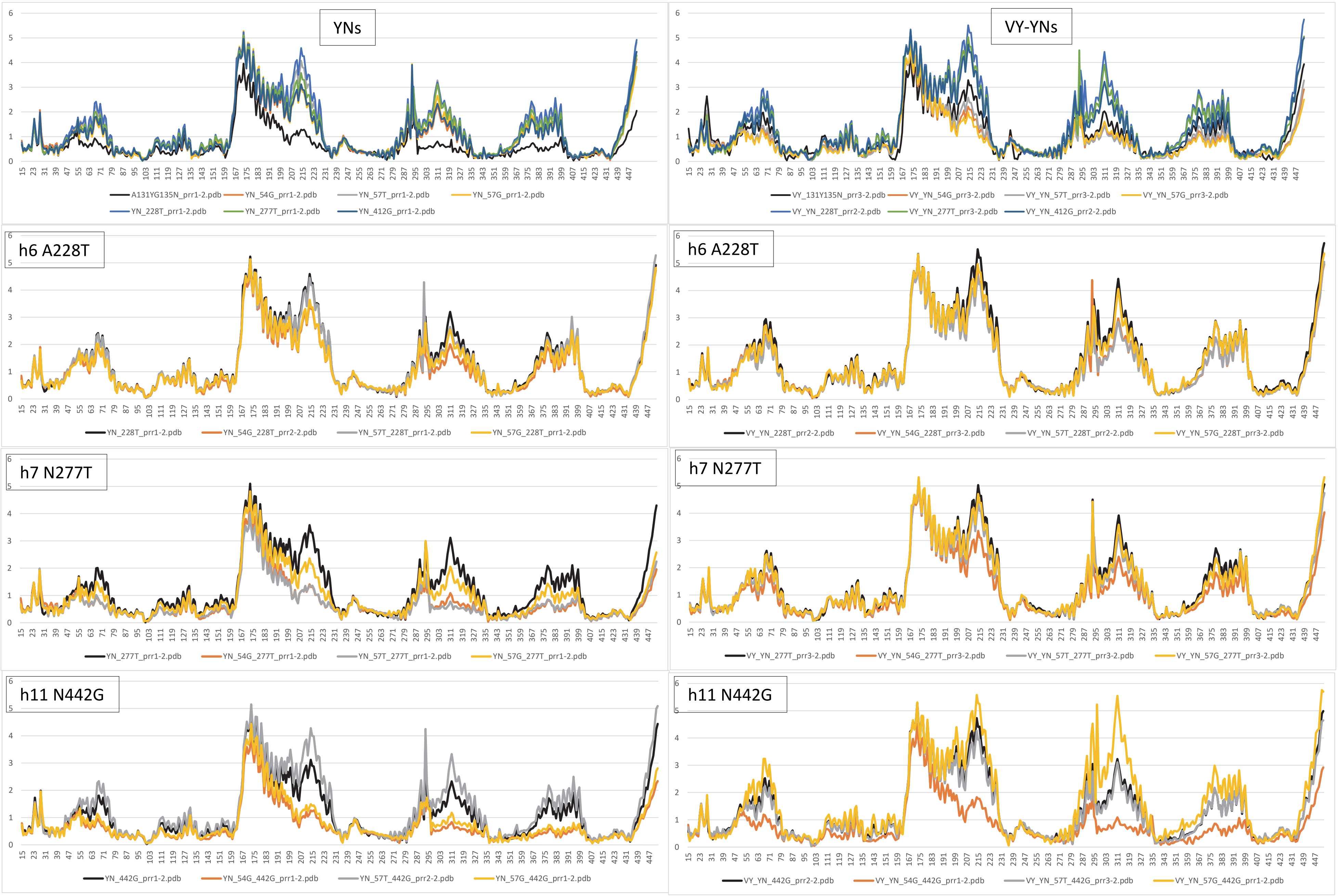

Figure S6. Conformational response to h1 Me^{2+} BS point mutations depends on Q5HQ64 genetic background. Per residue RMSD of Q5HQ64 mutants CF pdb model2 structurally aligned with wt Q5HQ64 CF pdb model4. None of the mutation tested individually influence wt Q5HQ64 conformation (data not shown). In YN background (*Left panel*) all mutations tested cooperate with YN, producing broad scale deviation (top), while the impact of h1 mutations (D54G, N57T and N57G) depends on other mutations in h6 (A228T), h7 (N277T) or h11 (N442G). In VY YN background (*Right panel*) h1 mutations limit overall deviation (top); yet their effect is minimal in the presence of h6 A228T, which produces maximal deviation, while D54G has negative impact in the presence of h7 N277T (mild) or h11 N412G (strong).

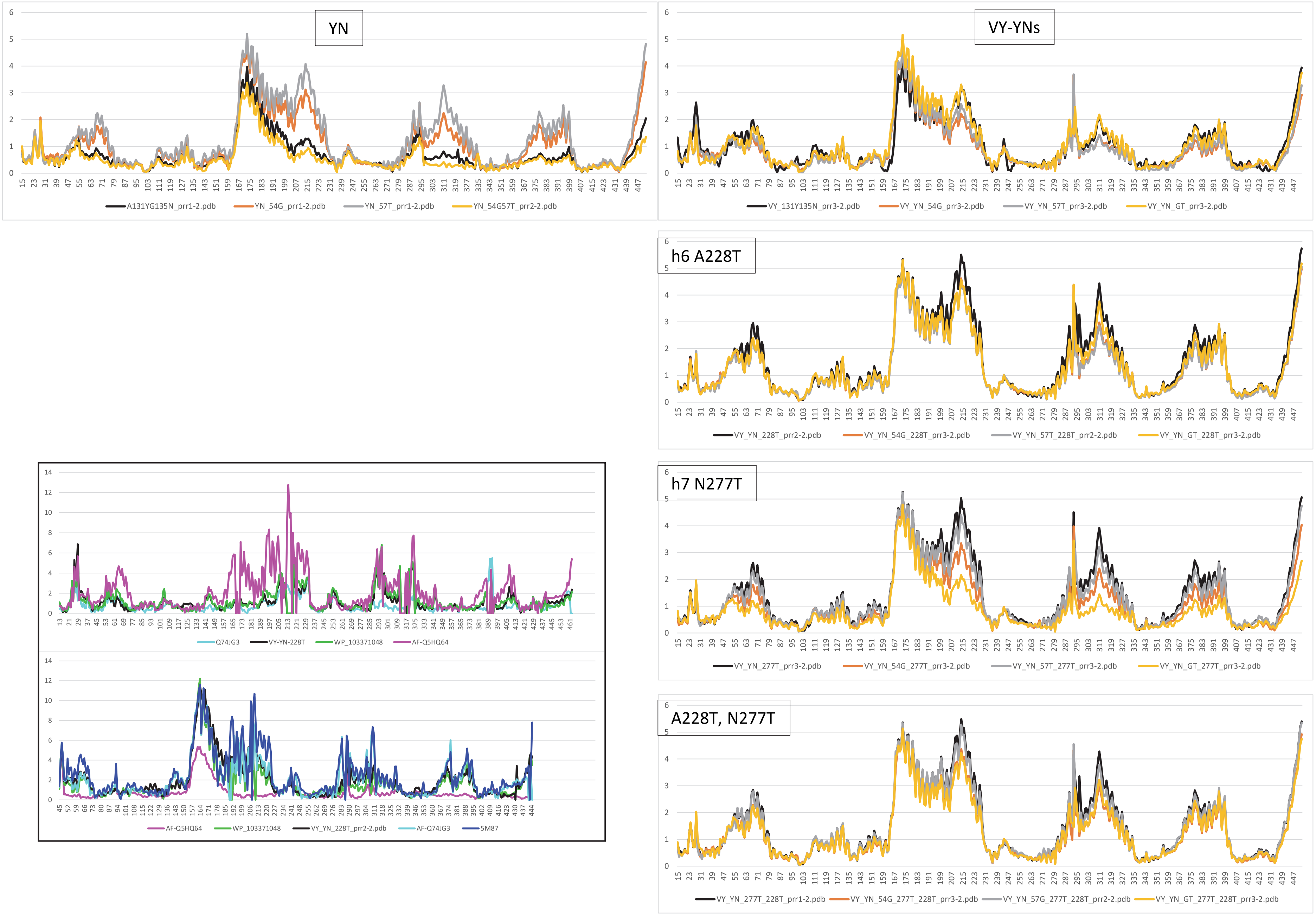

Figure S7. Mutations of Q5HQ64 h1a and h1b Me^{2+} BS have interdependent effects. *Left panel*. In h3 YN background, h1 D54G N57T dual mutation abrogates the cooperative effect of each of h1 mutation taken separately. *Right panel*. In h3 YN h6 VY background, h1 D54G N57T dual mutation has more positive effect than each mutation taken separately (top); it has minimal impact in the presence of h6 A228T background and exerts counter productive interaction with h7 N277T that is compensated by h6 A228T. *Inset*: Per residue RMSD of Q5HQ64 VY YN 228T (VNT) mutant compared to select AF2/CF MCb models (cf Figure 3) relative to both MCb reference structures (OO 5M87, top, and IO 5M94, bottom).

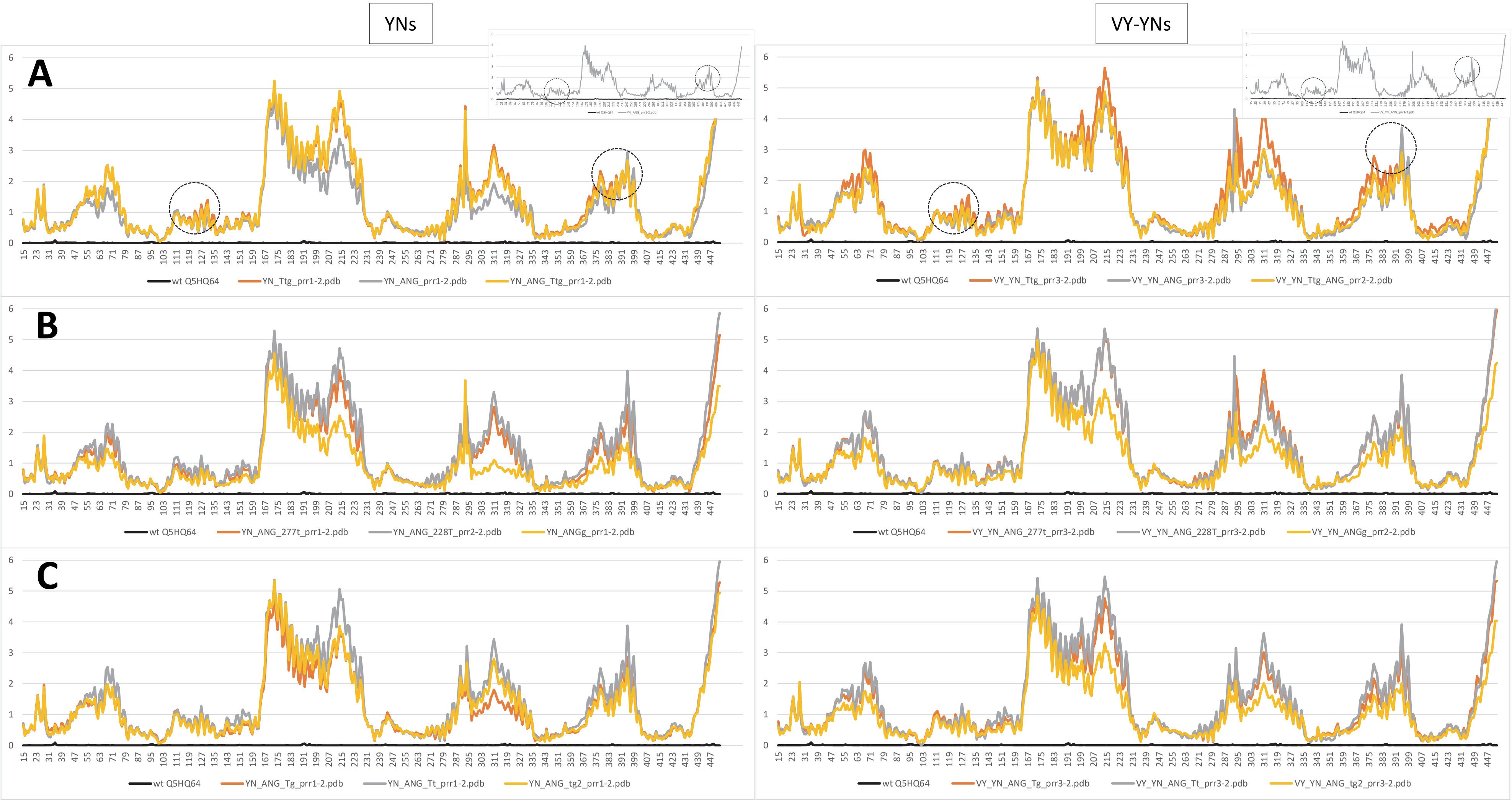

Figure S8. Evidence for direct interaction between MCb Q5HQ64 h3 and h10. Similar mutation combinations were tested both in YN background (*Left panel*) and VY YN background (*Right panel*). Neither h10 ANG nor the triple mutation Ttg were sufficient alone to produce broad scale deviation (data not shown). **A**. Effect of h10 triple mutation ANG (S393A L397N S398G, detailed in *insets*) compared to and combined with the triple mutation Ttg (h6 A228T, h7 N277T, h11 N442G). Circles indicate the location of h3 h10 inter helix contacts. **B**. h10 ANG combined to either h6 A228T, h7 N277T or h11 N442G. **C**. h10 ANG combined with either h6 A228T h11 N442G, h6 A228T h7 N277T or h7 N277T h11 N442G. (Baseline, re-run of wt Q5HQ64, model4).

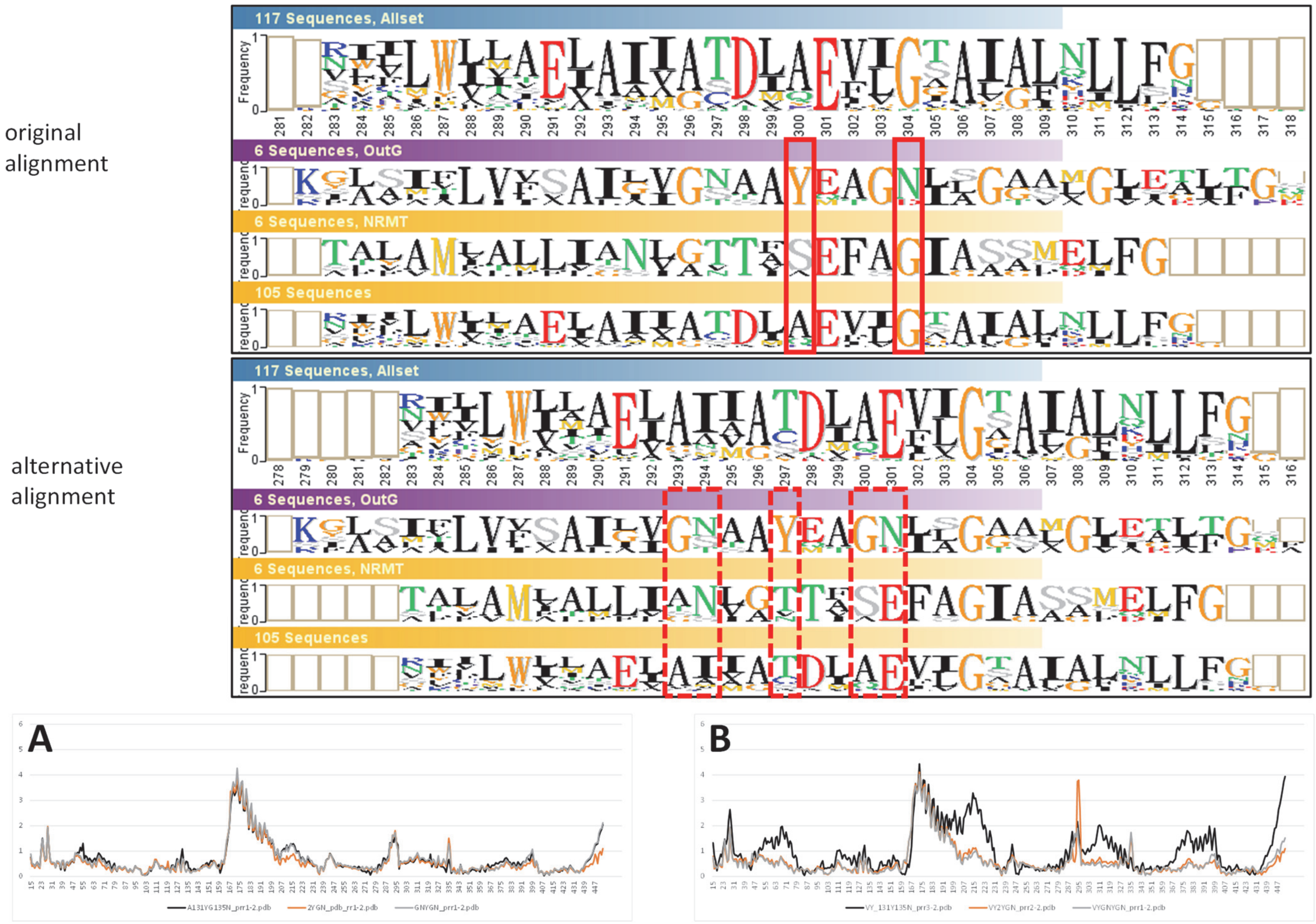

Figure S9. Testing h3 multiple sequence alignment and deduced selection of targeted sites for mutagenesis. *Top panel*. Comparison of alternative h3 alignments and deduced type ii evolutionary rate shifts targeted for mutagenesis (highlighted). The original h3 alignment was used to establish Slc11 phylogeny (cf Figure 1) and the alternative h3 alignment results from sliding both Slc11 and NRMT sequences to the right (+3). Bottom panel. Two combinations of mutations deduced from sites identified with the alternate alignment, h3 ----Y--GN and h3 GN--Y--GN, were introduced in Q5HQ64, either alone (**A**) or in combination with h6 VY (**B**) prior to CF pdb modeling and structural alignment with CF pdb model4.

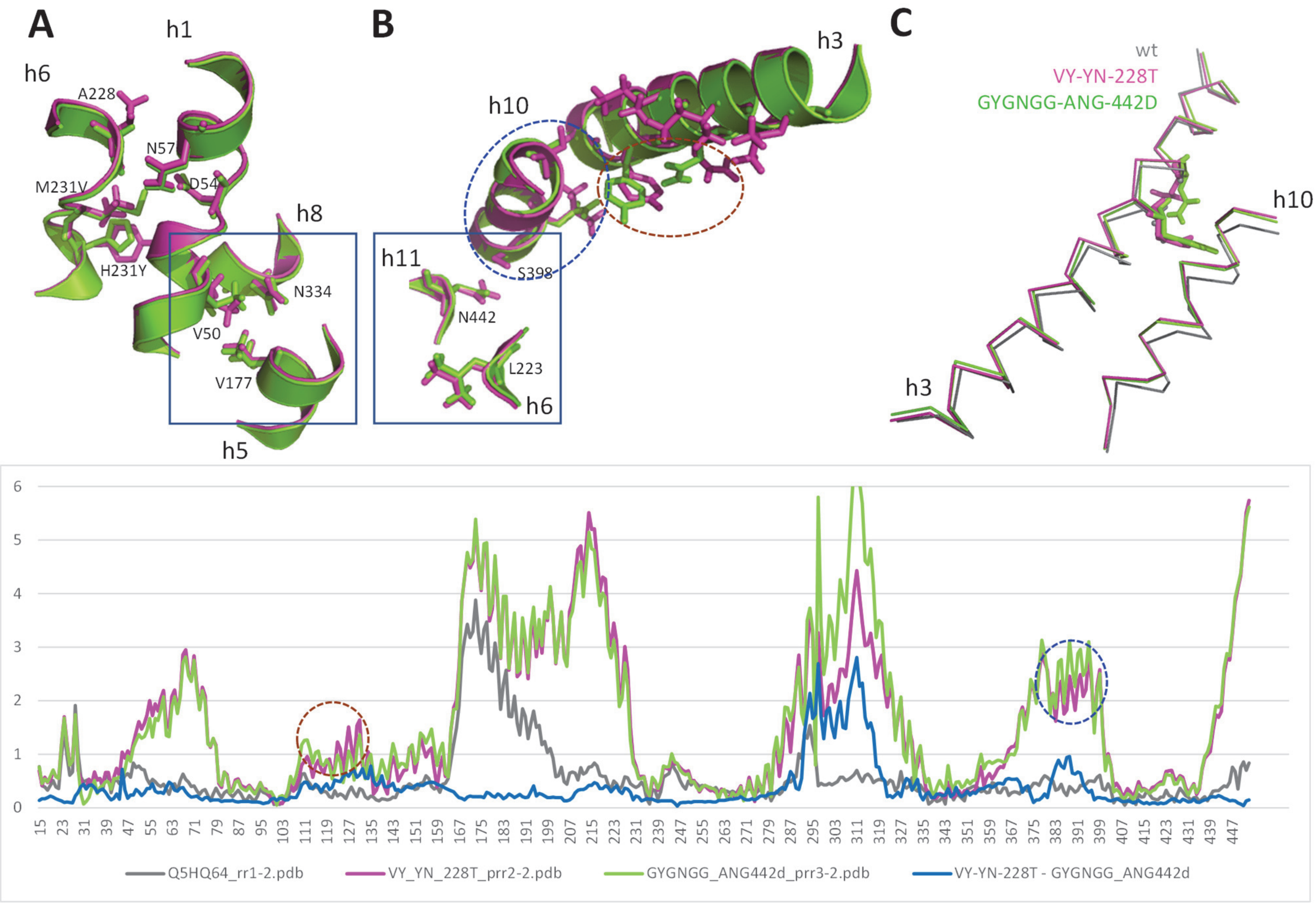

Figure S10. Accommodation of h3 YN mutation in Q5HQ64 compound mutants VY YN 228T and GYGNGG ANG N442D. *Top panel*. **A**. VY YN 228T and GYGNGG AND 442D mutants display superposable arrangements of the Me^{2+} BS and the OO-specific 5M87 community 7 of networked residues (boxed). **B**. The IO-specific 5M94 community 11 [nc18] of connected residues (boxed) also appears superposable while residue side chains in the h3 h10 contact area deviate. **C**. h3 h10 contact area. *Bottom panel*. Per residue RMSD of wt Q5HQ64, VY YN 228T and GYGNGG AND 442D mutants. The blue line shows pairwise deviation between VY-YN-228T and GYGNGG-ANG-442D models.

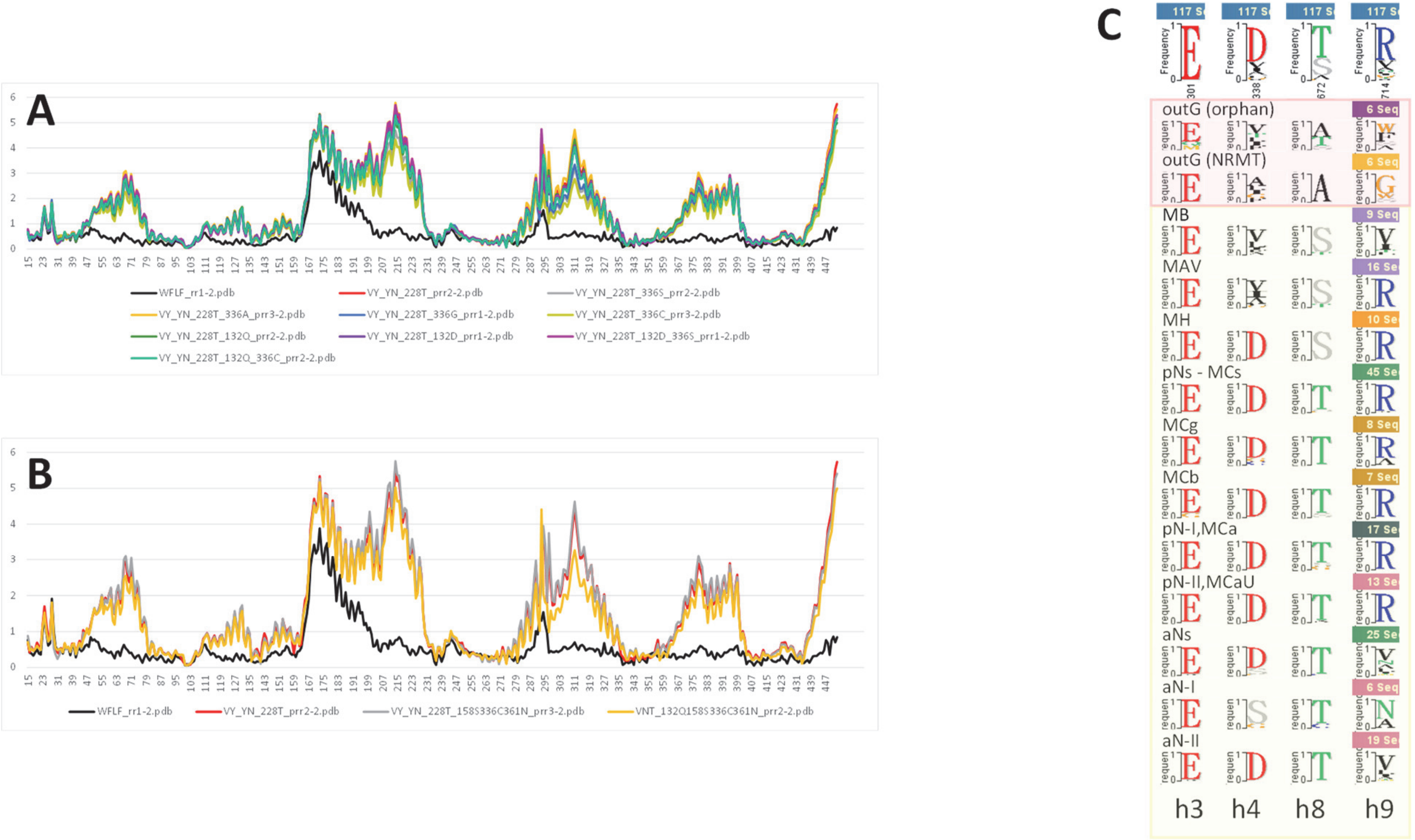

Figure S11. Effect of mutations targeting Slc11 H⁺-network on CF pdb modeling of Q5HQ64 VNT (VY YN A228T) mutant. **A**. Multiple substitutions of h8 T336 and h3 E132 alone or combined. **B**. Compound mutant h3 E132Q h8 T336C h9 R361N with or without h4 D158S. **C**. Natural evolutionary variation of each of the targeted sites.

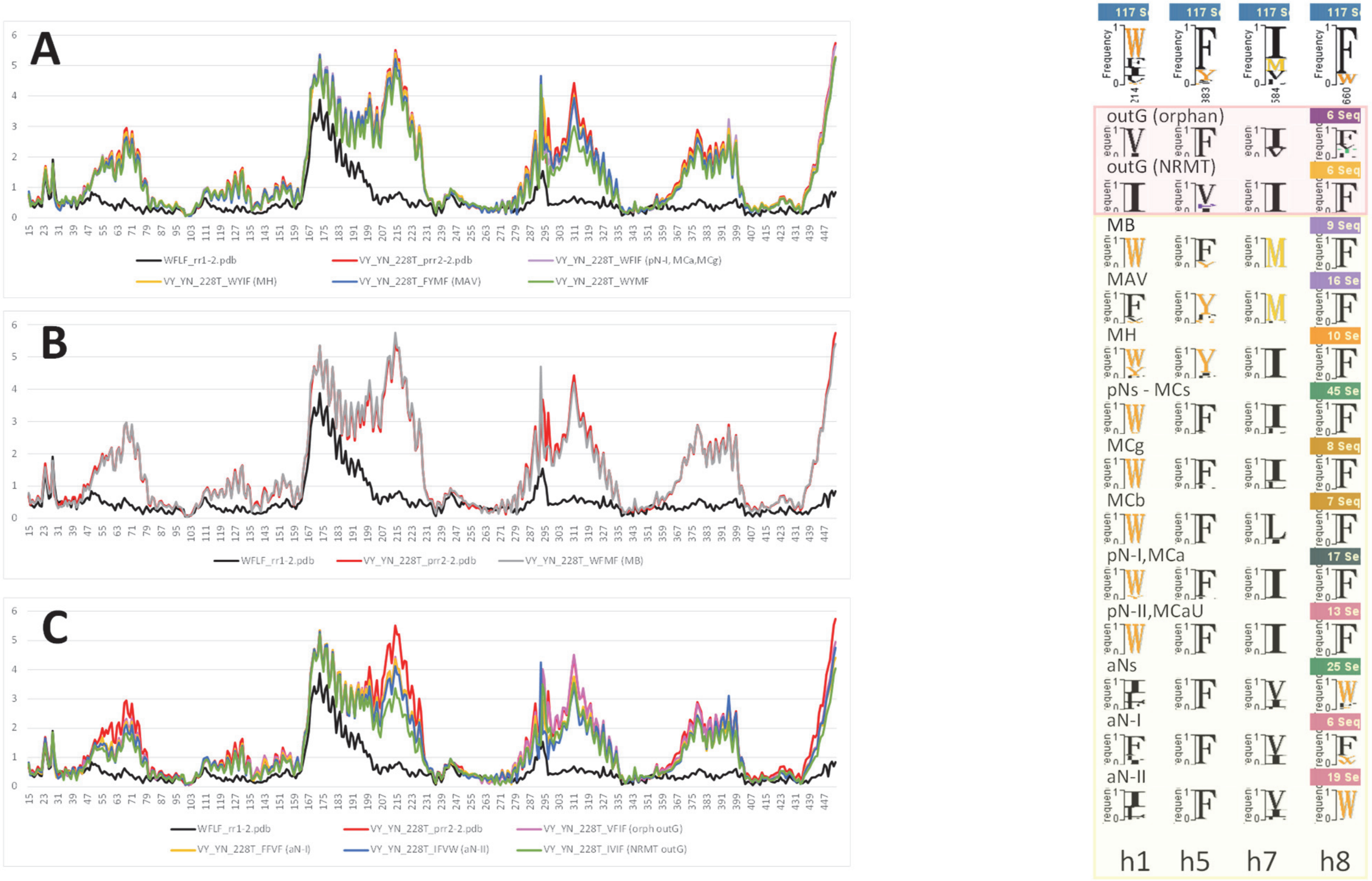

Figure S12. Interaction of h1b, h5, h7 and h8 may regulate coordinated motion of h1b-l1/2 and h6a in Q5HQ64 VY YN A228T. *Left panel.* Influence of mutations mimicking Slc11 clade selective consensus residue combinations on CF pdb modeling of Q5HQ64 VNT mutant. **A**. Consensus sequence for pN-I, MCa and MCg (WFIF); MH (WYIF); MA, MAV (FYMF) plus additional mutant WYMF. **B**. Consensus sequence for MB (WFMF). **C**. Consensus sequence for orphan outgroup (VFIF), NRMT outgroup (IVIF), aN-I (FFVF) and aN-II (IFVW). *Right panel*. Natural evolutionary variation of the targeted sites (i.e., Q5HQ64 h1 W58, h5 F188, h7 L280 and h8 F324).

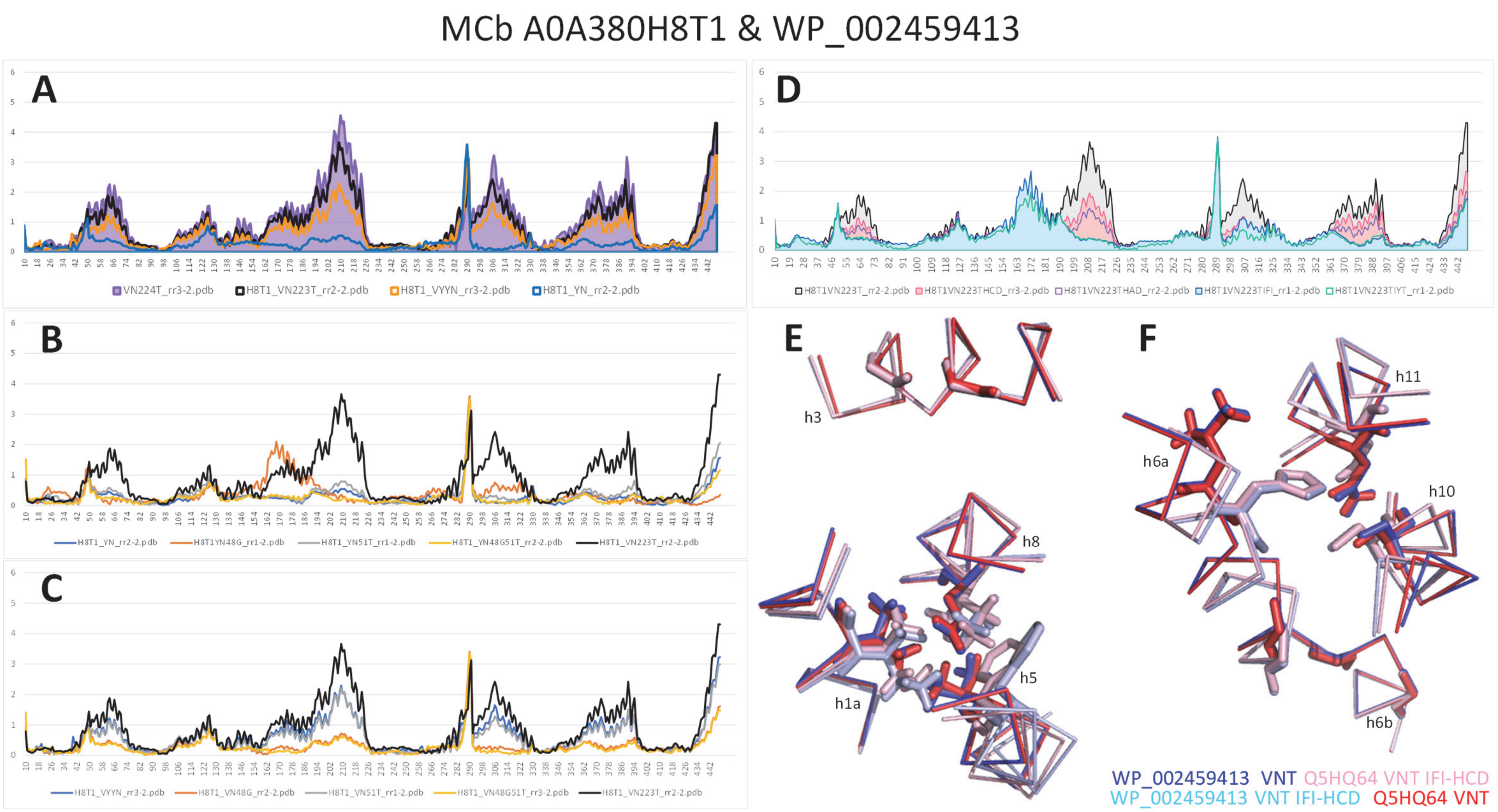

Figure S13. In silico mutagenesis of MCb gating communities prevents VNT-induced switch of CF pdb MCb models. **A-D**. Per residue RMSD from wt A0A380H8T1 or WP_002459413 IO conformer of models obtained for various mutants. **A**. Combined mutations inducing IO to OO conformation switch for both A0A380H8T1 (VNT, h3 YN h6 VY A223T) and WP_002459413 (VN224T). **B&C**. Combinations of A0A380H8T1 h1 mutations (D48G, N51T or both) with h3 YN background (**B**) or with h6 VY and h3 YN background (**C**). **D**. Separate targeting of either MCb OO community (5M87 c7: IFI, h1a V50I, h5 V177F, h8 N334I and IYT, h1a V50I, h5 V177Y, h8 N334T) or MCb IO community (5M94 c11 [nc18]: HCD, h6a L223H, h10 S398C, h11 N442D and HSD, h6a L223H, h10 S398A, h11 N442D) in A0A380H8T1. **E&F**. Combined targeting of both communities (OO community, inner gate, IFI, and IO community, outer gate, HCD) in WP_002459413. Superposed models obtained for VNT (dark color) and VNT IFI-HCD (light color) mutants of both WP_002459413 (blue) and Q5HQ64 (red).

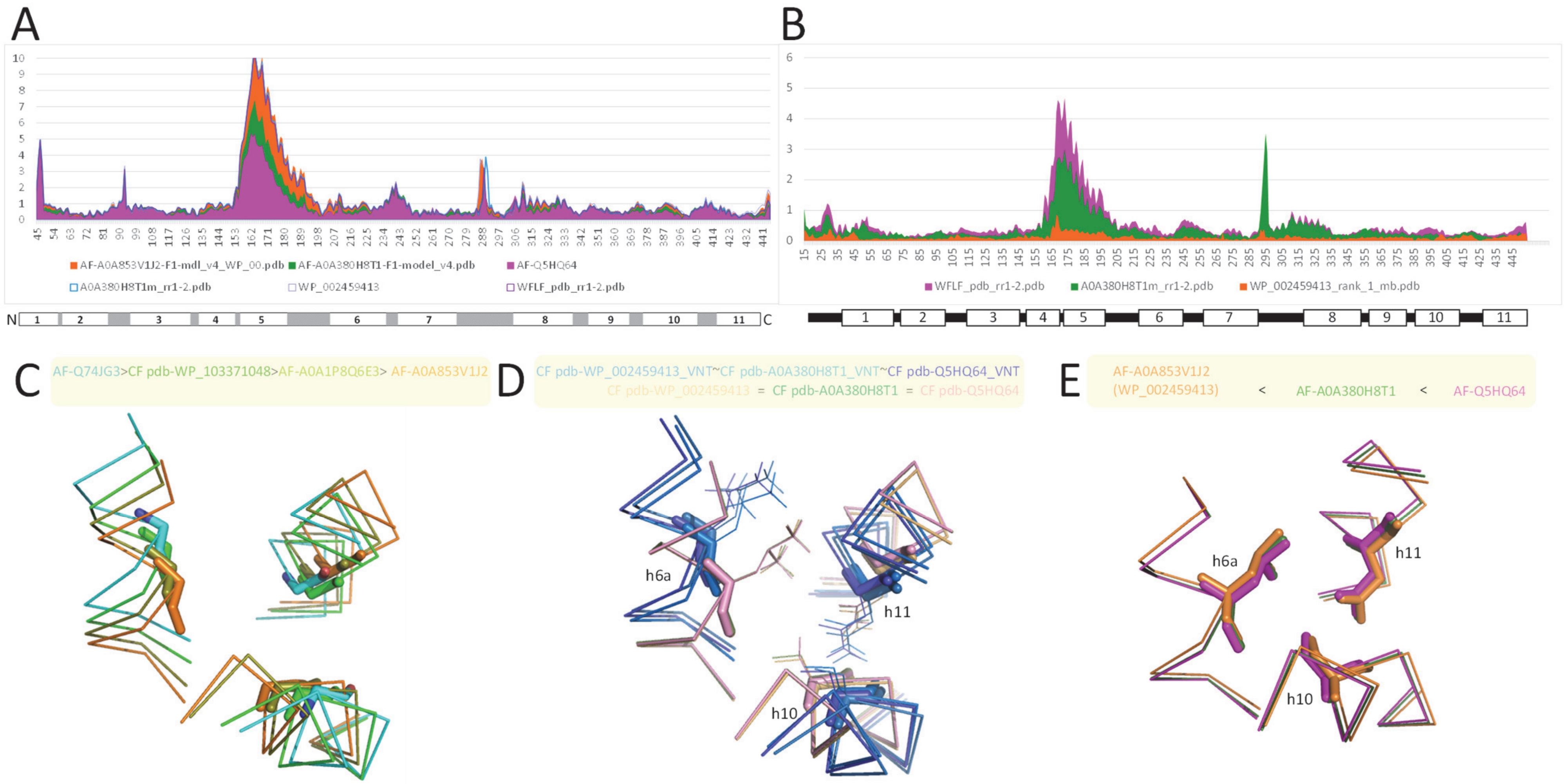

Figure S14. MCb forward transition (OO to IO) comprises two successive processes distinguished by AF2 vs CF pdb modeling. A. *Per residue RMSD from MCb 5M94 IO structure of AF2 and CF pdb models for A0A853V1J2/WP_002459413, A0A380H8T1 and Q5HQ64).* ***B.*** *Respective per residue RMSD between pairs of AF2 and CF pdb models (A0A853V1J2/WP_002459413; A0A380H8T1; Q5HQ64).* ***C-I****. 3D superpositions showing evolution of local areas of MCb models during separate processes of OO carrier switch coupled to unlocking the inner gate (****C&G****), which is mimicked using the VNT mutation (****D&H****), vs inner gate opening throughout IO state (****E&I, F****). Accessions and corresponding color codes are indicated for each panel.*

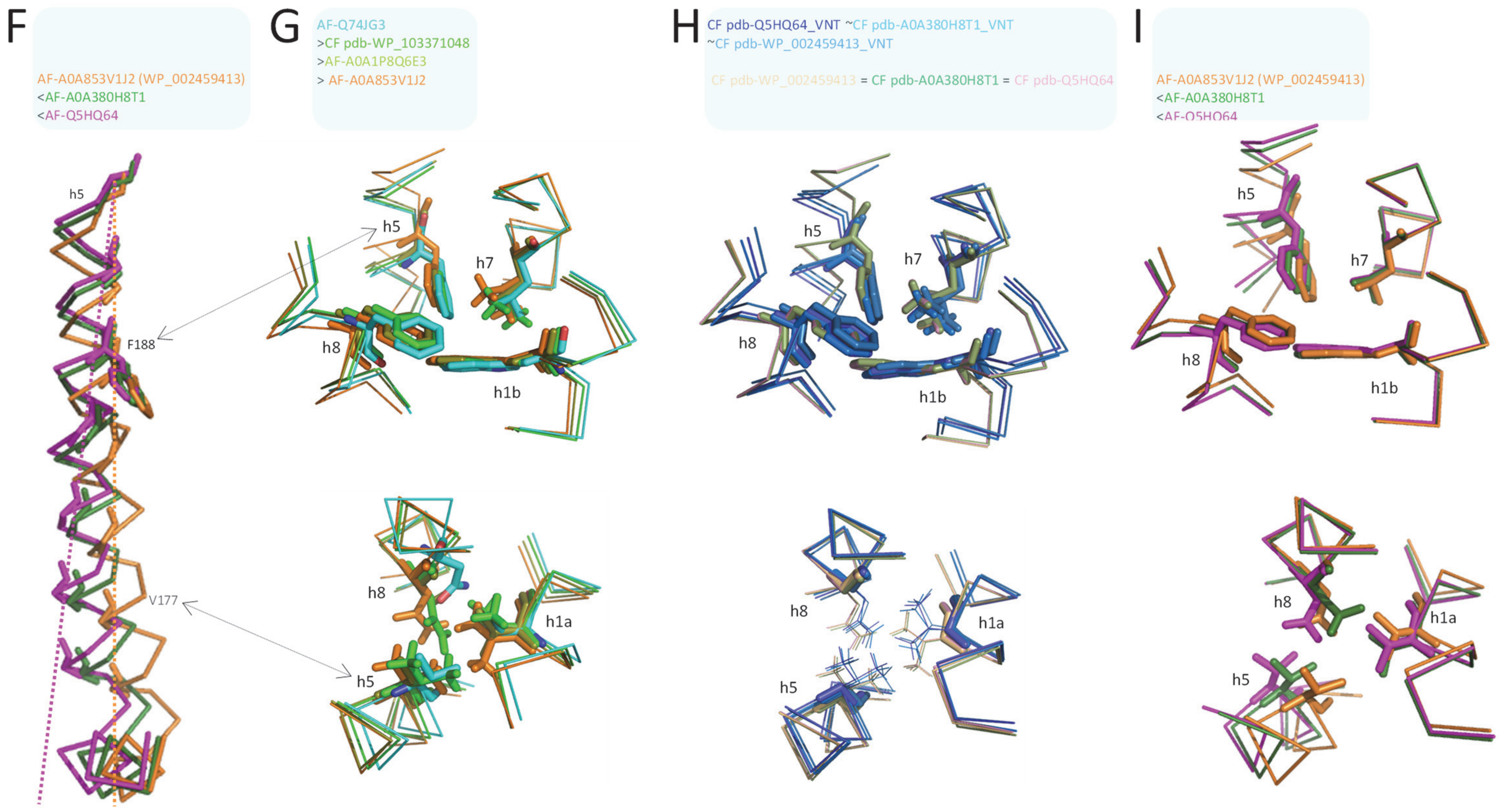

Figure S14 (contin'd).

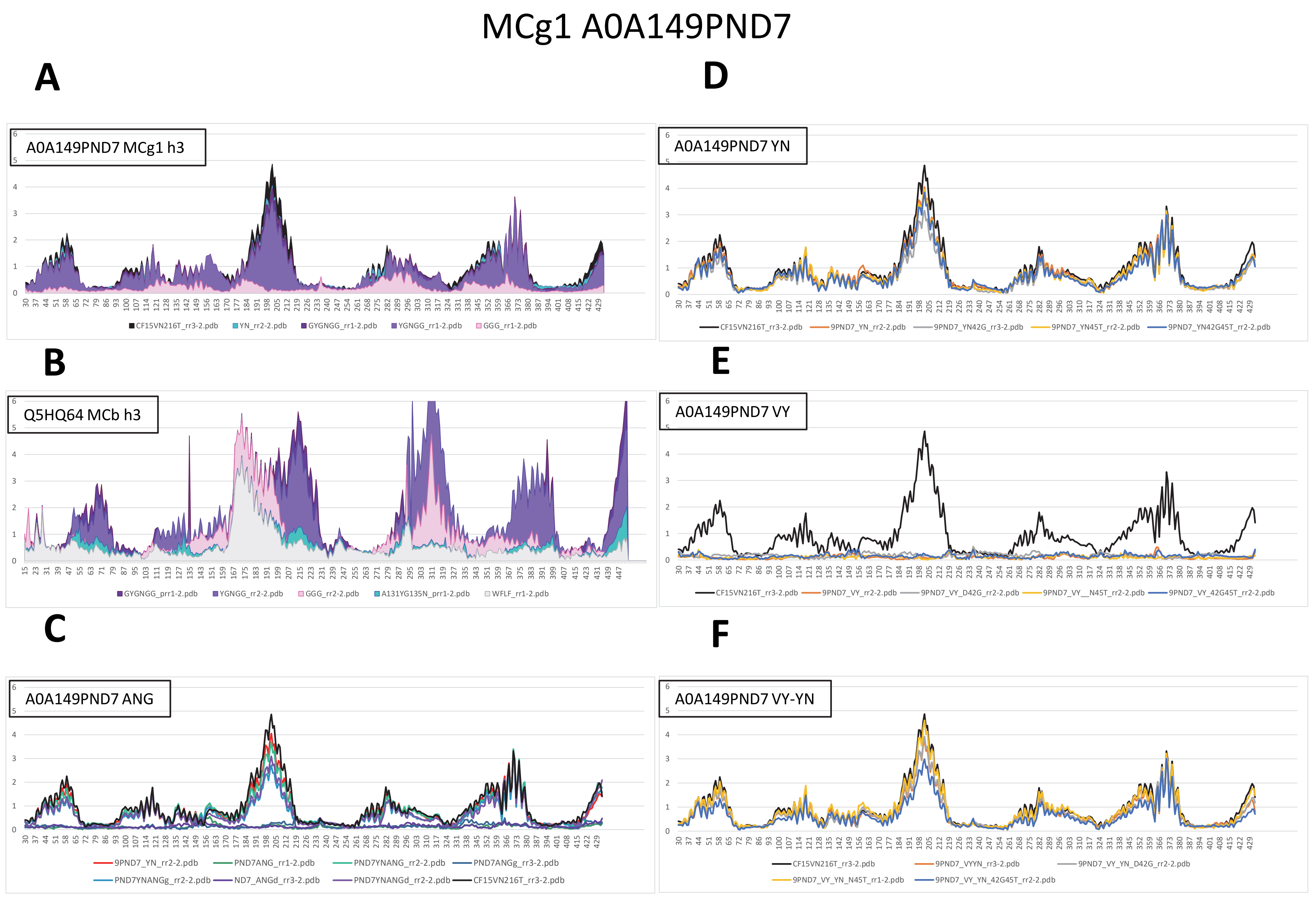

Figure S15. h3 YN mutation switching MCg1 A0A149PND7 IO conformation back to OO state is modulated by other synapormorphic sites (h1, h6, h10 and h11). Per residue RMSD for models obtained using CF nt or CF pdb and aligned with either wt A0A149PND7 CF nt model (**A**, **C**-**F**) or wt Q5HQ64 CF pdb model (**B**), respectively. **A, B**. Compound mutants h3 GGG,YN, YGNGG, GYGNGG of MCg1 A0A149PND7 (**A**) and MCb Q5HQ64 (**B**). **C**. h3/h10/h11 compound mutants ANG, YNANG, ANGg, YNANGg, ANGd, YNANGd. **D-F**. Mutation combinations of h3 YN (D), h6 VY (E) and h3 YN h6 VY (F) with h1 mutants D42G or N45T or both.

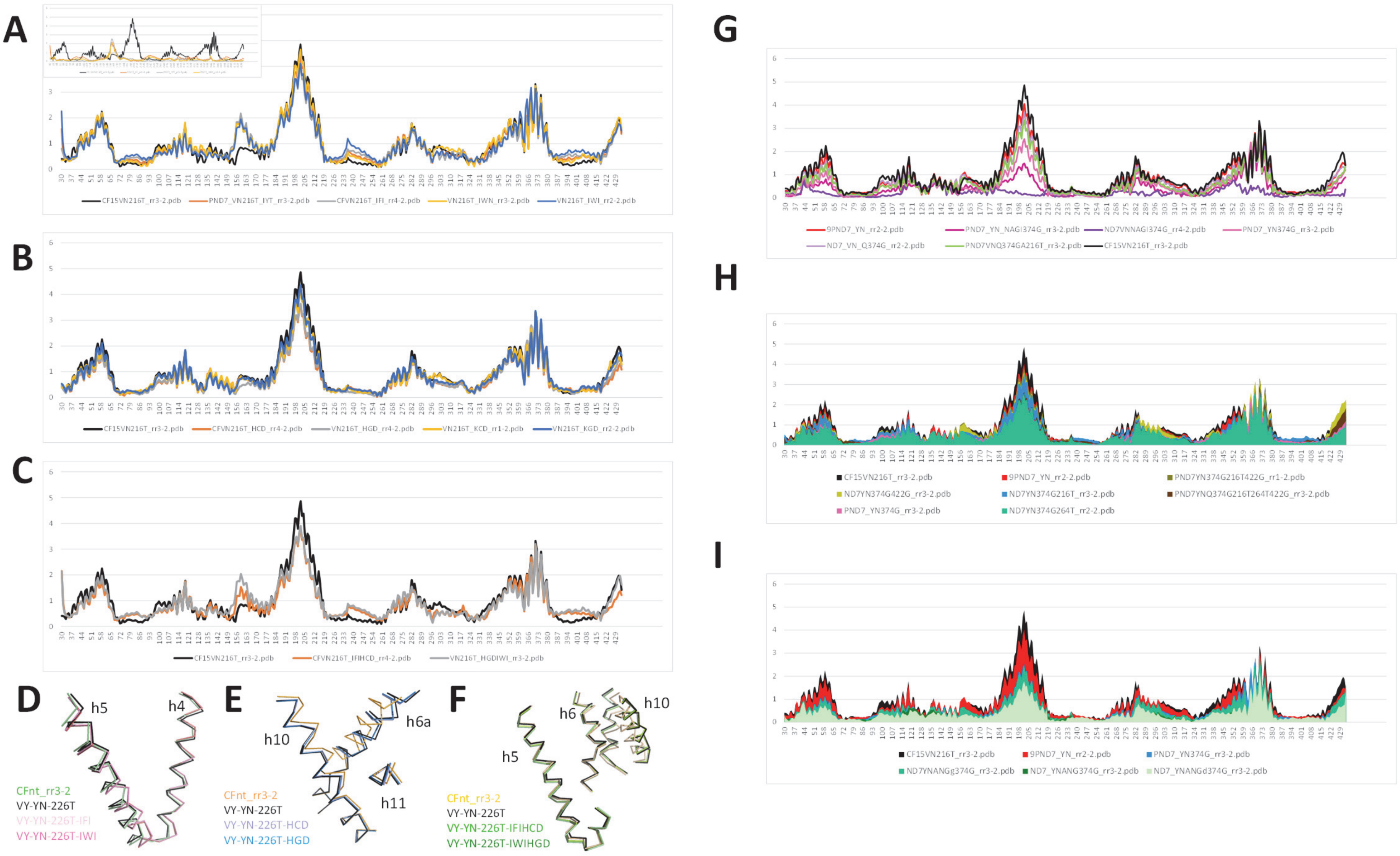

Figure S16. Structural divergence of MCg1 A0A149PND7 from MCbs. Per residue RMSD for models obtained using CF nt and aligned with wt MCg1 A0A149PND7 (CF nt) (**A-C**, **G-I**) are presented together with select Pymol Ca trace displays of POSA multiple alignment of MCg1 models (**D-F**). **A-C**. A0A149PND7 VNT mutant served to test the impact of mutations that prevented MCb IO to OO switch by altering carrier gating (outer gate, **A&D**, inner gate, **B&E,** both, **C&F**). **G-I**. A0A149PND7 h10 Q374G mutation suppressing YN-induced conformation switch allows probing cooperation with synapomorphic sites located in h6 (**G**), h7 and h11 (**H**), h10 and h11 (**I**).

MCg1 D4XFA5

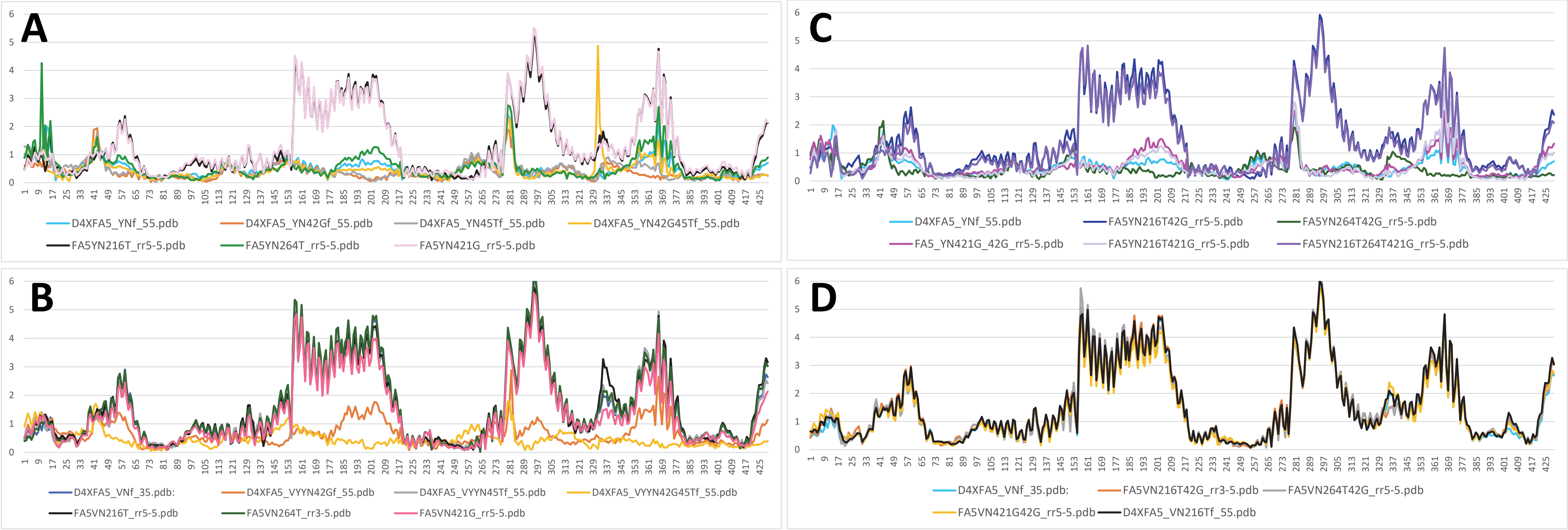

Figure S17. Influence of Slc11 synapomorphic site mutagenesis on MCg1 D4XFA5 conformation switch. Per residue RMSD of CFnt models for D4XFA5 mutants aligned with wt D4XFA5 CF nt rr3-5 used as reference IO structure (inner gate open) in h3 YN mutant background (**A&C**) or in h3 YN h6 VY mutant background (**B&D**). **A&B**. Effect of point mutations in h1 (D42G, N45T, 42G45T), h6 (A216T), h7 (N264T), h11 (D421G). **C&D**. Impact of h1 D42G mutation combined with h6 (A216T), h7 (N264T), h11 (D421G), and modulation of YN A216T D421G by N264T mutation (**C**).

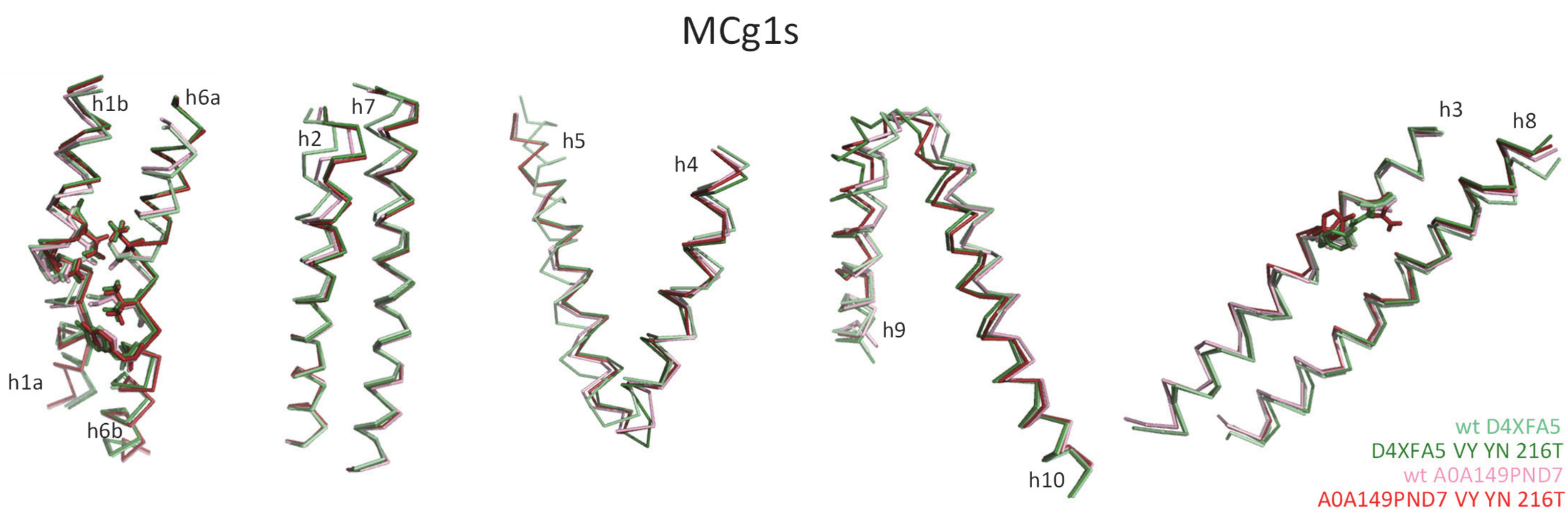

Figure S18. Similar switch of IO to OO conformers among divergent MCg1s. Two pairs of native MCg1 IO and VNT mutation-induced OO conformers (D4XFA5 and A0A149PND7) were superposed and Ca trace deviations are presented separately for either adjacent or linked helices. Sticks indicate residues forming the substrate binding site (h1 & h6) and the conformation-driving mutation h3 YN.

A

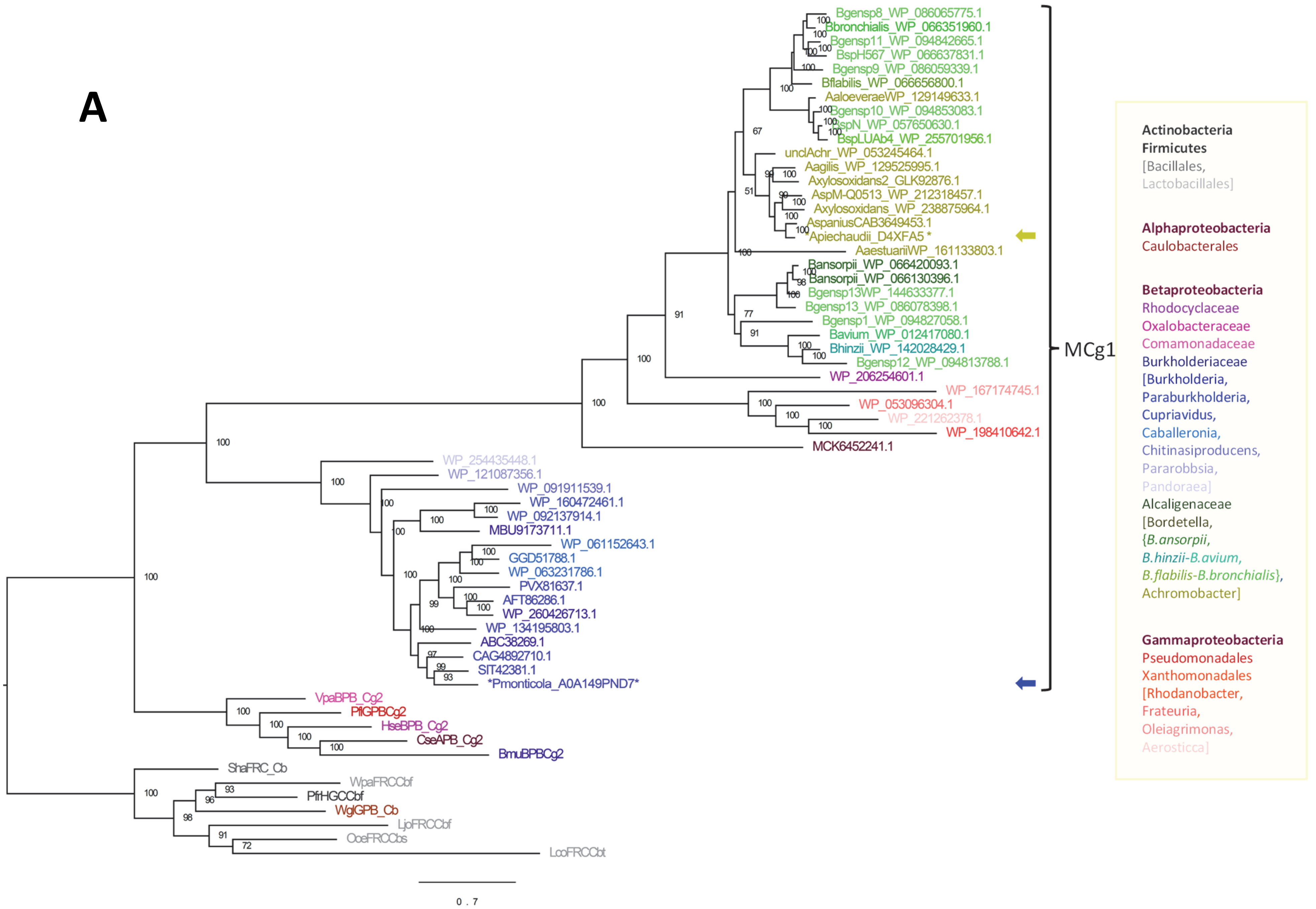

Figure S19. Phylogeny of MCg1. The IQ-trees presented were calculated using parsimony informative (PI) sites, the substitution mixture model EX-EHO, ML estimate of a.a. state frequency, free rate model of variation among sites with 10 (**A**) or 8 (**B**) categories. Also, 1000 replica of each of two types of branch support calculations (ultrafast bootstrap and SH-aLRT single branch test) were performed to estimate confidence in the tree clades obtained. A scale bar representing the number of substitutions per site is provided. **A.** IQ-Tree using 343 PI sites representing 61 seqs, including 32 seqs ("D4XFA5 MCg1 cluster"), 17 seqs ("A0A149PND7 MCg1 cluster"), 5 MCg2 seqs and seven MCb seqs, the latter being used to root the tree. The taxonomic distribution of the sequence encoding genomes is color-coded. The relative positions of D4XFA5 and A0A149PND7 in MCg1 phylogeny are indicated with arrows.

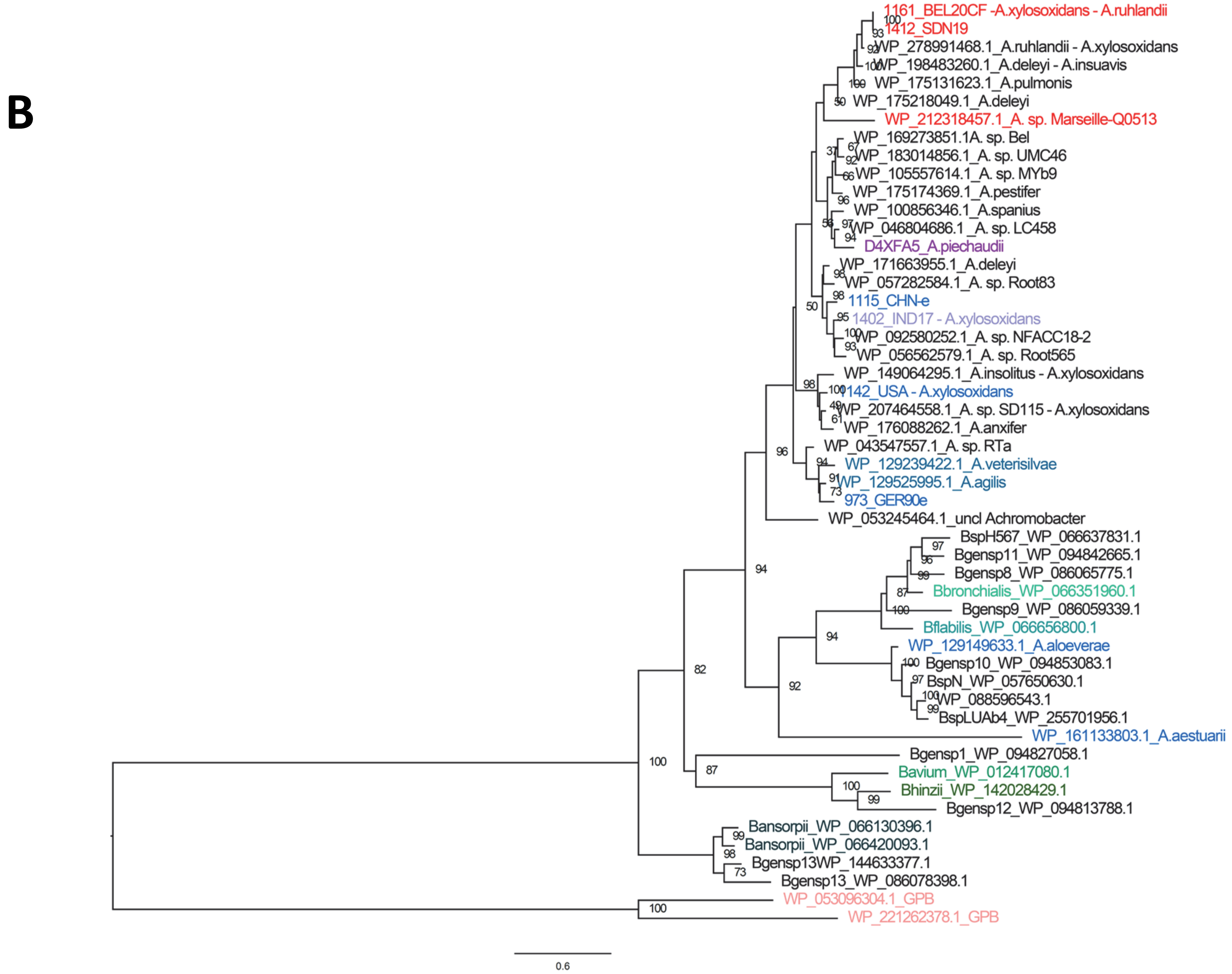

***Figure S19. Phylogeny of MCg1.* B.** IQ-Tree using 248 PI sites representing 51 seqs from D4XFA5 MCg1 cluster. Some of the most divergent seqs from this cluster (from GPB, cf A.) were used to root the tree (highlighted with salmon color). Seqs encoded by *Bordetella* spp. are indicated with green tints (*B. ansorpii; B. hinzi* & *B. avium*; *B. flabilis* & *B. bronchialis*). Different colors distinguish *Achromobacter* spp. of environmental origin (blue to violet tints) from those isolated from patients (red), including six reference strains from the PubMLST collection (1161_BEL20CF, 1412_SDN19, 1115_CHN-e, 1402_IND17, 1142_USA, 973_GER90e). The *Achromobacter* seqs used represent clusters (95% id, 13 singletons). One representative spp. name is provided; additional presence per cluster of isolates from either *A. xylosoxidans*, *A. ruhlandii* or *A. insuavis* is also indicated (potential emerging pathogens in patients with cystic fibrosis, CF).

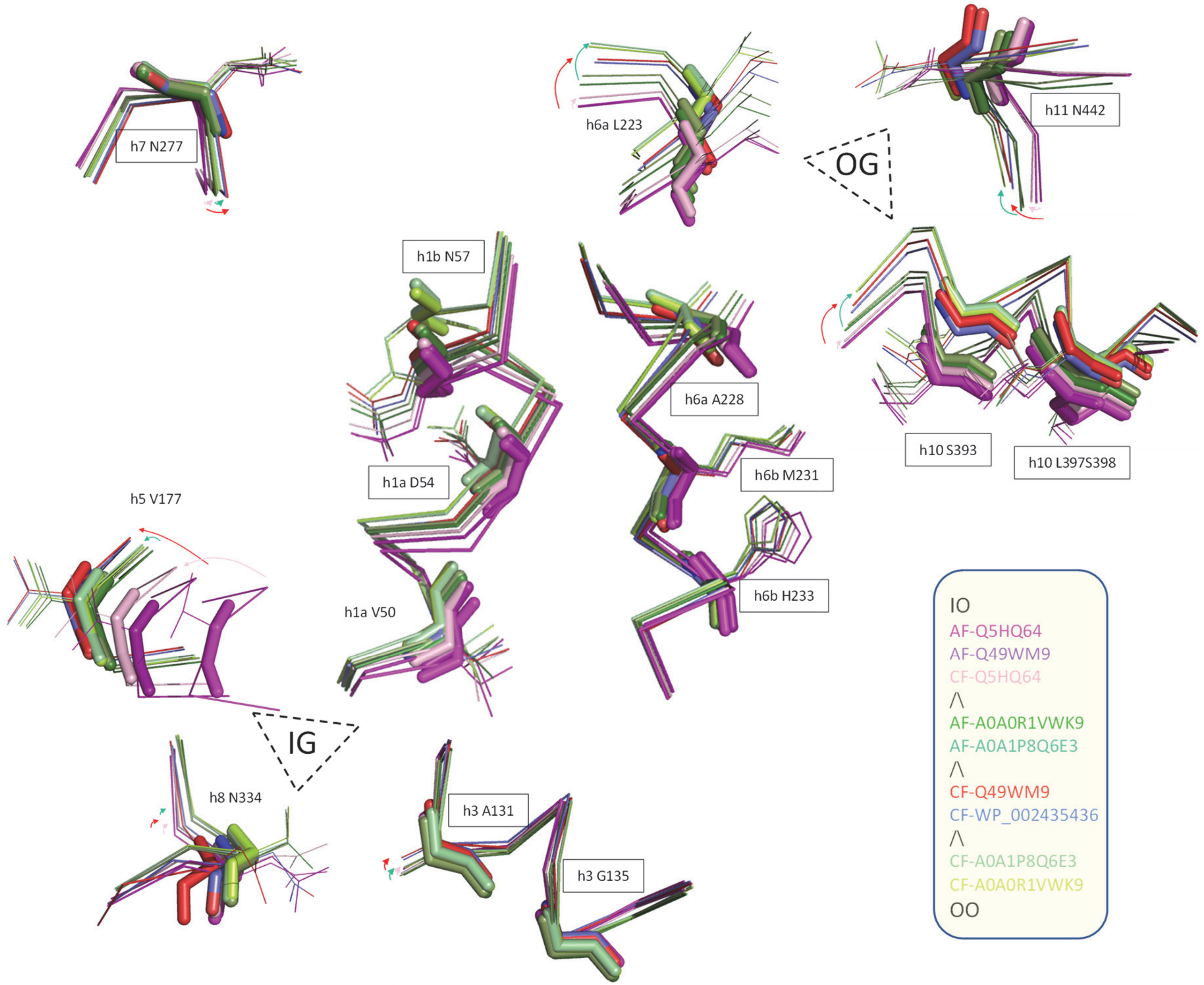

Figure S20. MCb modeling shows strong phylogenetic component. 3D superposition of the models listed as sequential intermediates spanning MCb carrier transition from OO to IO. The residues shown are represented as stick (main chain) and line (side chain, no H) and colored as per model. The residues forming Slc11 synapomorphy are boxed; other residues presented contribute to outer or inner gating (h6a and h1a, h5, h8, respectively) as part of networked communities schematized as dotted triangles. Colored arrows indicate main chain displacement between respective AF2 and CF pdb models.
