# Supplementary figures and images for: Slc11 Synapomorphy: A Conserved 3D Framework Articulating Carrier Conformation Switch
Source: Int J Mol Sci. 2023 Oct 11;24(20):15076. doi: 10.3390/ijms242015076 (PMC10606218; doi:10.3390/ijms242015076)

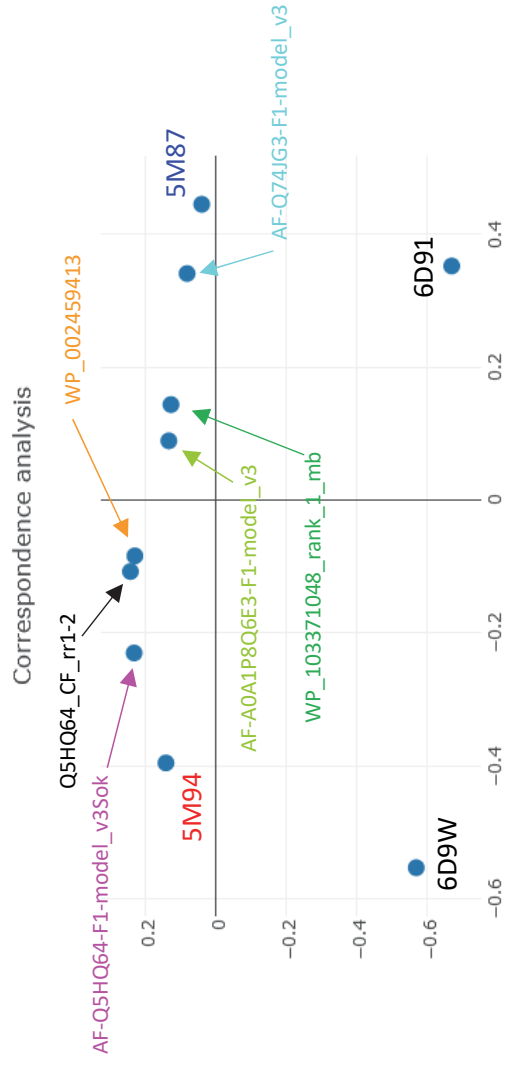

MCb

MA

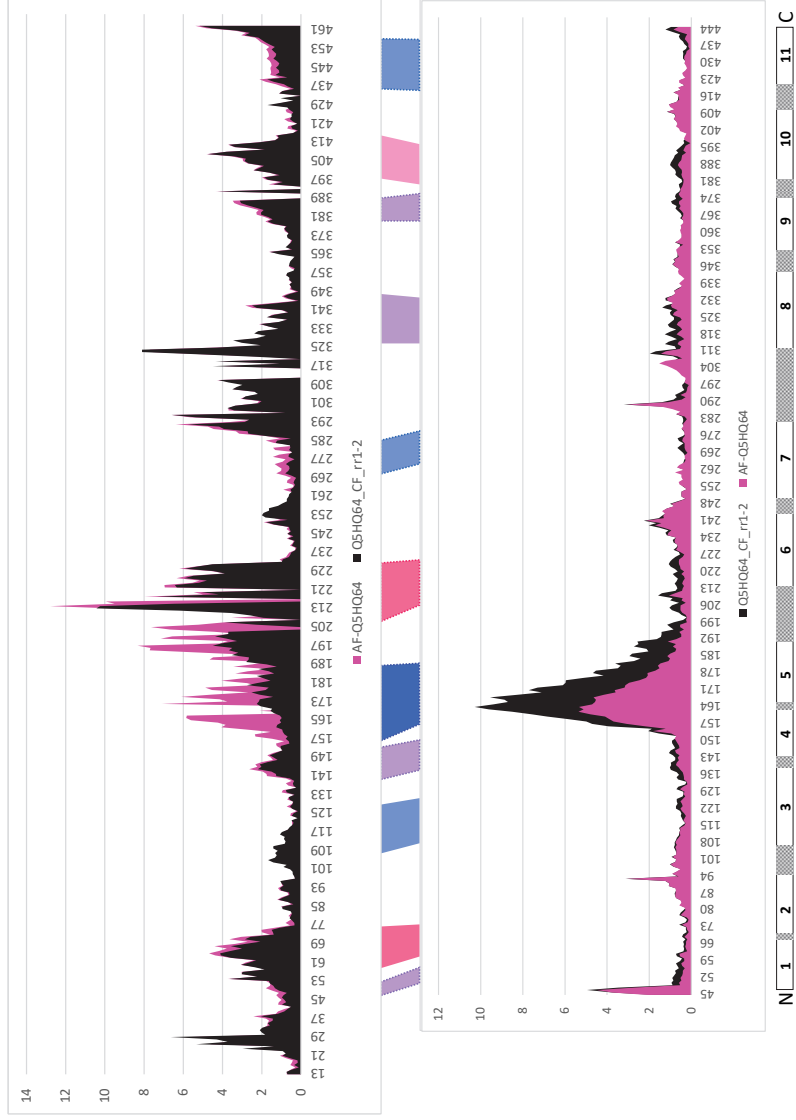

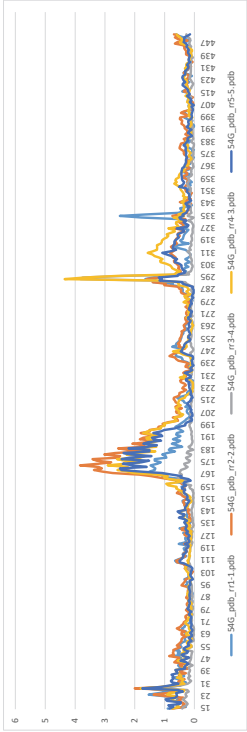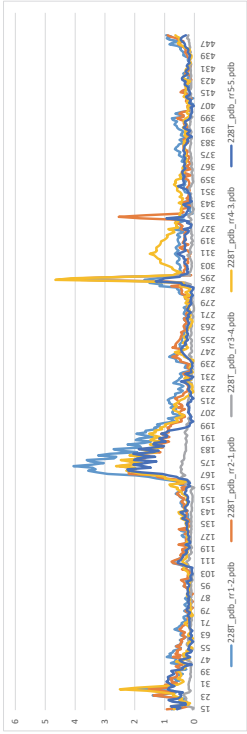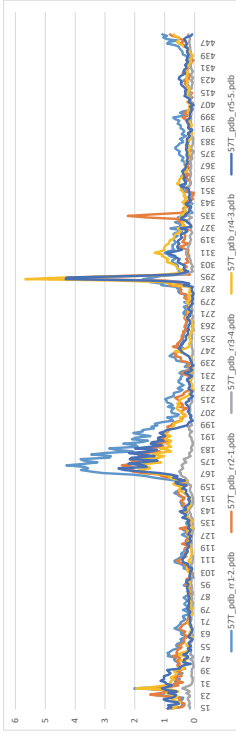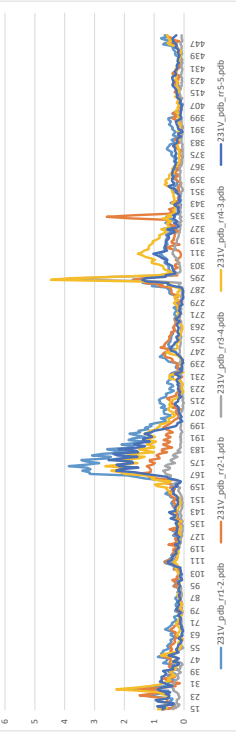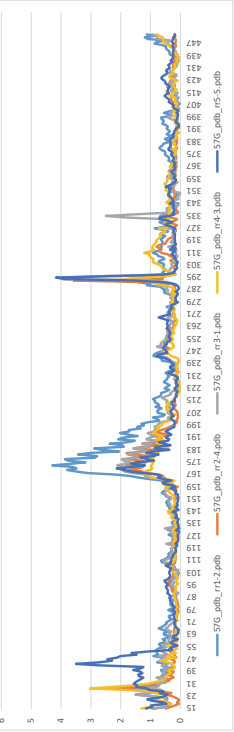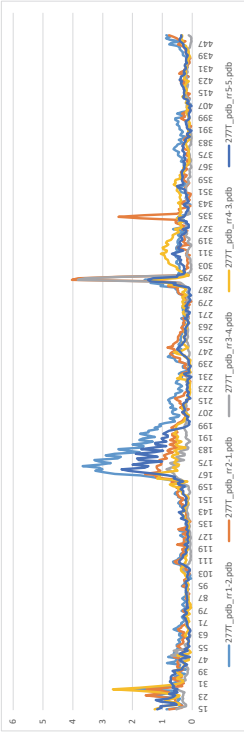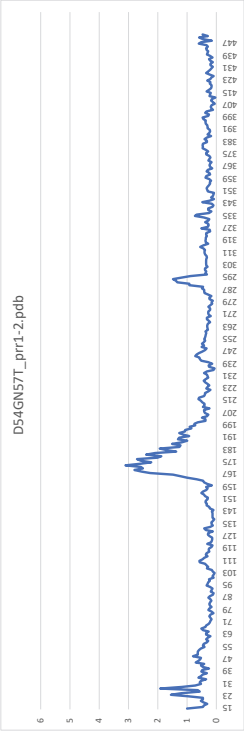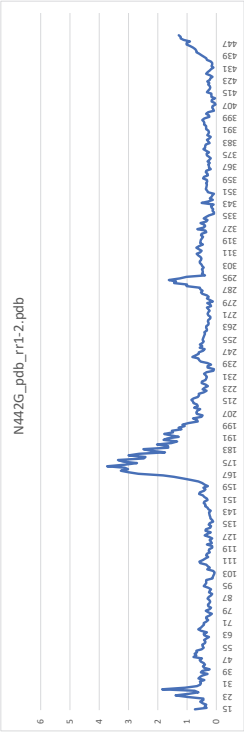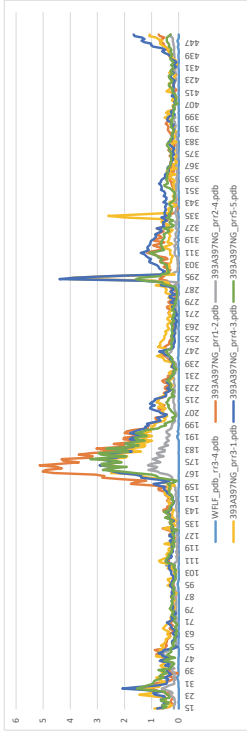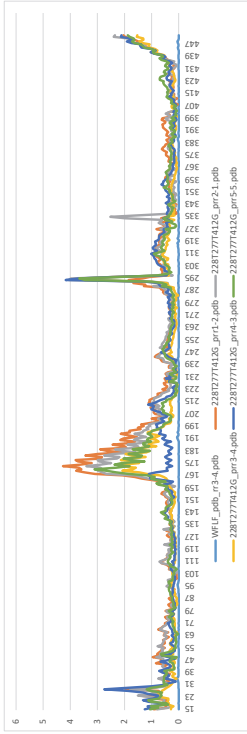

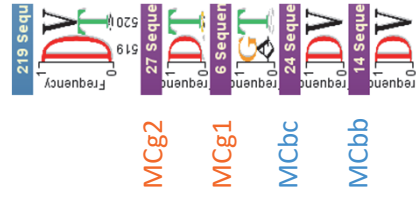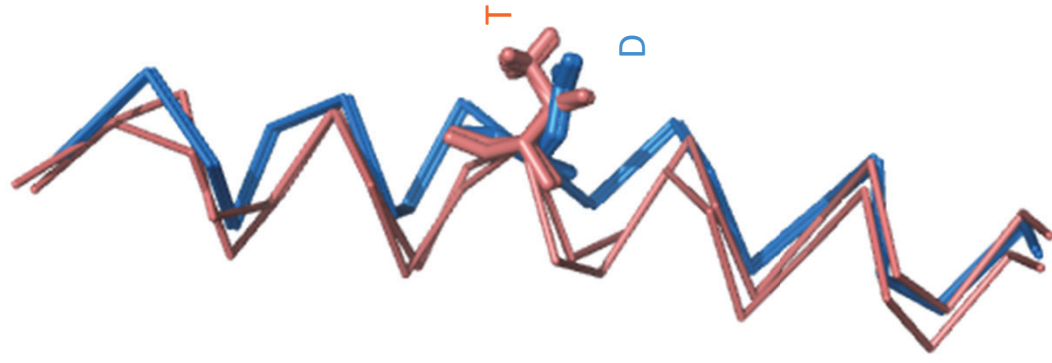

Native MCg1 h4

Native MCb h4

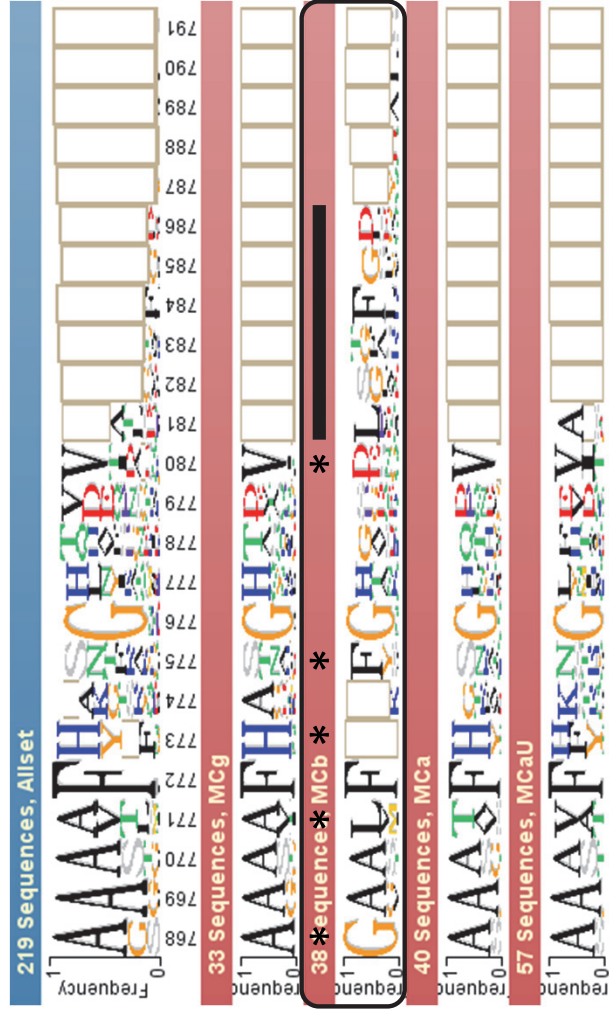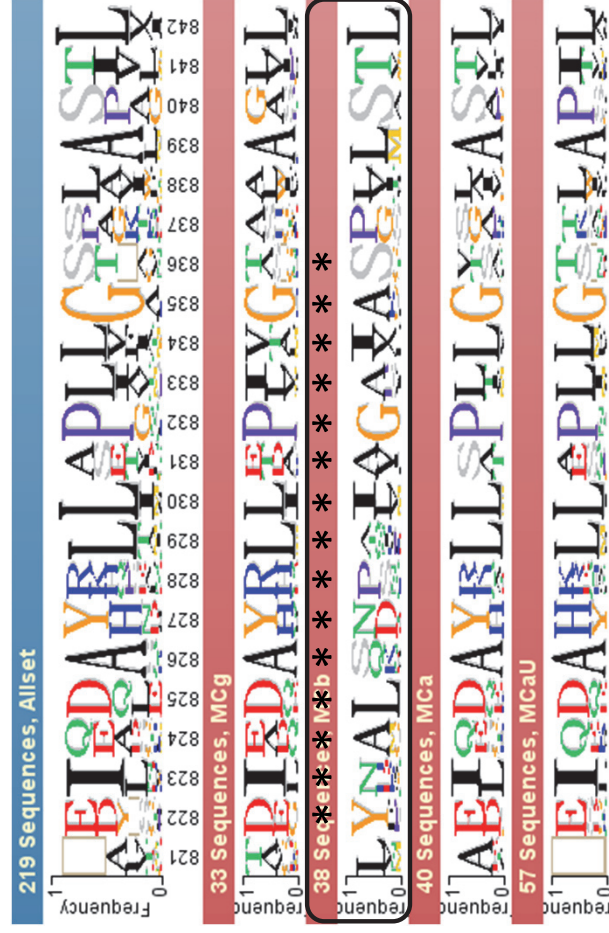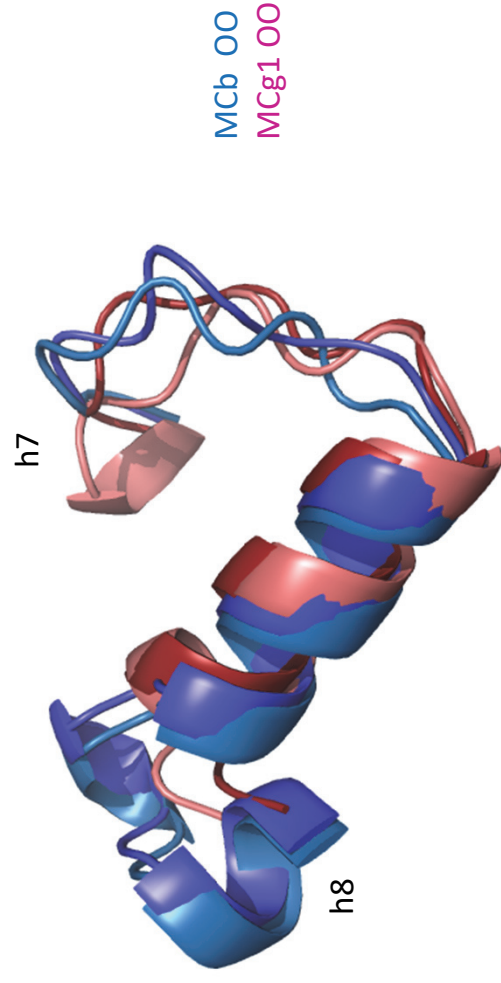

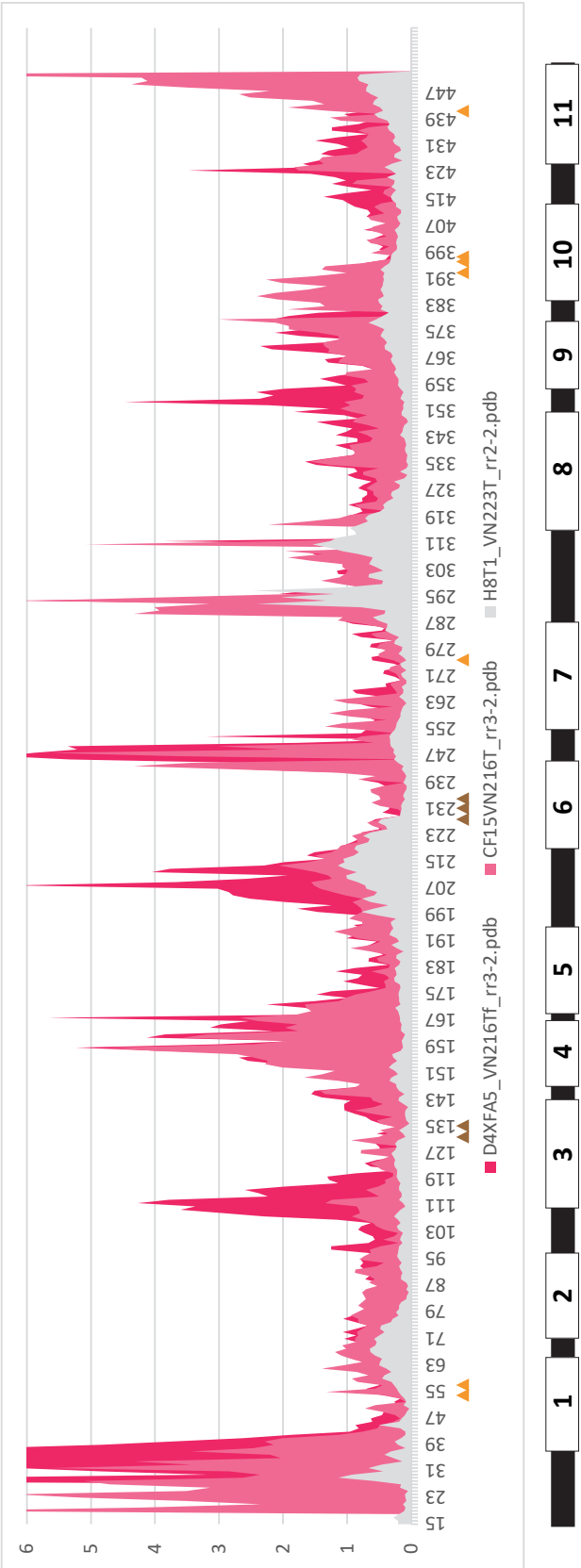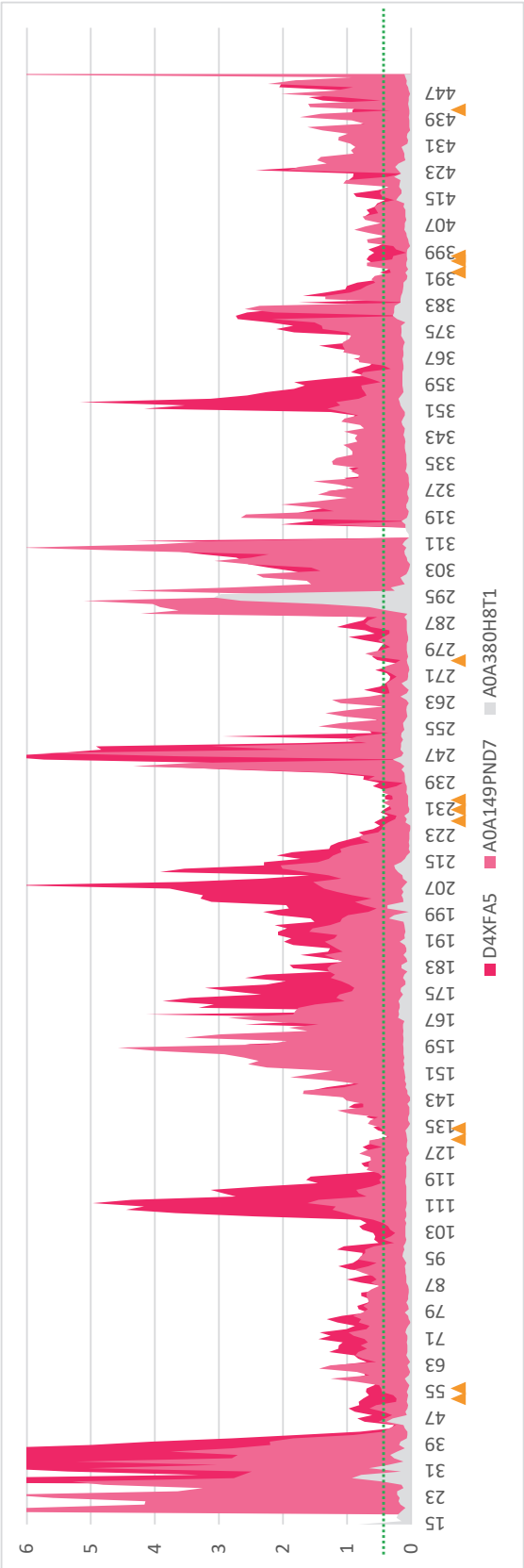

Supplement: Supplementary file 1 [file ijms-24-15076-s001.zip › supplementary file S1-ijms-2587053-non-published data1-5.pdf]
